# Low‐Temperature Processed Efficient and Reproducible Blade‐Coating Organic Photovoltaic Devices with *γ*‐Position Branched Inner Side Chains‐Containing Nonfullerene Acceptor

**DOI:** 10.1002/smsc.202400034

**Published:** 2024-04-15

**Authors:** Donghoo Won, So‐Huei Kang, Jaeyeong Park, Jeewon Park, Wonjun Kim, Thi Le Huyen Mai, Seunglok Lee, Changduk Yang

**Affiliations:** ^1^ School of Energy and Chemical Engineering Perovtronics Research Center Low Dimensional Carbon Materials Center Ulsan National Institute of Science and Technology (UNIST) 50 UNIST‐gil, Ulju‐gun Ulsan 44919 South Korea; ^2^ Graduate School of Carbon Neutrality Ulsan National Institute of Science and Technology (UNIST) 50 UNIST‐gil, Ulju‐gun Ulsan 44919 South Korea

**Keywords:** blade coating, film uniformity, low‐temperature processing, reproducibility, solubility

## Abstract

Recent advancements in blade‐coating organic photovoltaic (OPV) devices utilizing eco‐friendly nonhalogenated solvents have demonstrated high power conversion efficiencies (PCEs) when processed at high substrate temperatures. However, this method poses challenges in device reproducibility and stability. Herein, a BTP‐eC9‐*γ* nonfullerene acceptor (analogous to BTP‐eC9) with *γ*‐position‐branched inner side chains within the BTP‐eC9‐based structural motif is developed. This pin‐sized extension in the branching position enhances the solubility of BTP‐eC9‐*γ* in nonhalogenated toluene solvent. This improvement not only mitigates excessive aggregation in the film state but also facilitates device fabrication at lower substrate temperatures. Optimized at a substrate temperature of 40 °C, the BTP‐eC9‐*γ*‐based blade‐coating devices with toluene achieve remarkable PCEs of 16.43% (0.04 cm^2^) and 14.95% (1.0 cm^2^). Furthermore, these devices retain their high film uniformity at 40 °C, which contributes to superior device reproducibility. This is attributed to the minimized alteration in the evolution kinetics of fluid flow. These findings signify a promising direction for the industrial production of blade‐coating OPV devices.

## Graphical abstract

This study addresses the reproducibility and film uniformity challenges of blade coating by implementing lower processing temperatures. The branched inner side chain of the renowned acceptor BTP‐eC9 to enhance solubility is extended. This modified acceptor demonstrates successful blade coating at lower substrate temperature (40 °C), achieving power conversion efficiencies of 16.43% (0.04 cm^2^) and 14.95% (1.0 cm^2^).

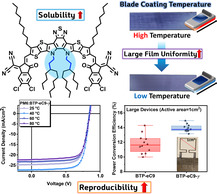

## 1 Introduction

The rapid advancement of nonfullerene acceptors, characterized by their exceptional absorption properties and tunable energetics, has propelled power conversion efficiencies (PCEs) up to 20% in small‐area single‐junction organic photovoltaic (OPV) devices.^[^ ^1^ ^]^ State‐of‐the‐art OPV devices are predominantly fabricated using a spin‐coating technique that employs environmentally harmful halogenated solvents.^[^ ^2^ ^]^ Notably, the spin‐coating process often results in significant PCE reduction when translating from small to large‐scale OPV production, attributable to nonuniform film thickness caused by distinct forces at the center and edges of the substrate.^[^ ^3^ ^]^ This inconsistency presents significant challenges for industrial‐scale OPV device manufacturing.^[^ ^4^ ^]^

Among various printing techniques developed to address the limitations of spin‐coating, blade‐coating serves as a foundational tool for slot‐die coating and roll‐to‐roll printing processes; it has emerged as a cost‐effective and high‐throughput technology for achieving satisfactory performance in large‐area OPV devices.^[^ ^5^ ^]^ In particular, controlling crystallization through surface tension design and blade‐coating conditions during the blade‐coating wetting process is crucial for producing uniform, high‐quality films, enabling to guarantee of high‐performance OPV devices.^[^ ^6^ ^]^ Compared to the spin‐coating process, the slower solvent evaporation duration of blade‐coating facilitates active material crystallization, often resulting in the growth of larger aggregations/crystals—a factor contributing to the PCE deterioration.^[^ ^7^ ^]^ Consequently, the substrate temperature becomes a critical factor in achieving the desired blend morphology during the blade‐coating process.^[^ ^8^ ^]^

Numerous recent examples of high‐performance blade‐coating OPV devices utilizing environmentally benign nonhalogenated solvents have been processed at elevated substrate temperatures.^[^ ^9^ ^]^ This approach is adopted to achieve optimal blend morphology in the active materials, ensuring uniformity, and appropriate phase‐separation domains.^[^ ^10^ ^]^ However, high substrate temperatures can adversely affect blend morphology, device reproducibility, and stability. Consequently, the development of efficient blade‐coating OPV devices that combine low substrate temperatures with nonhalogenated solvents is crucial for their practical and industrial application.

In our research, we modified the well‐known BTP‐eC9 nonfullerene acceptor by shifting the branching position of the inner side chains from the β‐position to the *γ*‐position. This modification yielded the synthesis of a novel BTP‐eC9‐*γ*, which exhibits a larger electrochemical bandgap and blue‐shifted absorbance compared to BTP‐eC9. Thereafter, we investigated its application in blade‐coating OPV devices. The *γ*‐position‐branched inner side chains enhance solubility in nonhalogenated toluene solvent as well as prevent excessive aggregation in the film state.^[^ ^11^ ^]^ This improvement facilitates efficient blade‐coating OPV fabrication at low substrate temperatures, ensuring adequate processability, and film quality with optimal miscibility.^[^ ^12^ ^]^

When blended with PM6 donor polymer in single‐junction OPV construction, BTP‐eC9‐*γ*‐based blade‐coating devices processed at 40 °C with toluene achieved impressive PCEs of 16.43% (0.04 cm^2^) and 14.95% (1.0 cm^2^). These efficiencies notably surpass those of their BTP‐eC9‐based counterparts, which were optimized at a higher substrate temperature of 80 °C. Most importantly, the BTP‐eC9‐*γ*‐based blade‐coating devices at 40 °C demonstrated exceptional reproducibility with minimal PCE deviations across both small‐ and large‐area OPV devices. This consistency is attributed to minor changes in the morphology evolution kinetics of fluid flow and is a crucial factor for commercial applications.

## 2 Results and Discussion

### 2.1 Synthesis and Characterization

**Figure 1:**
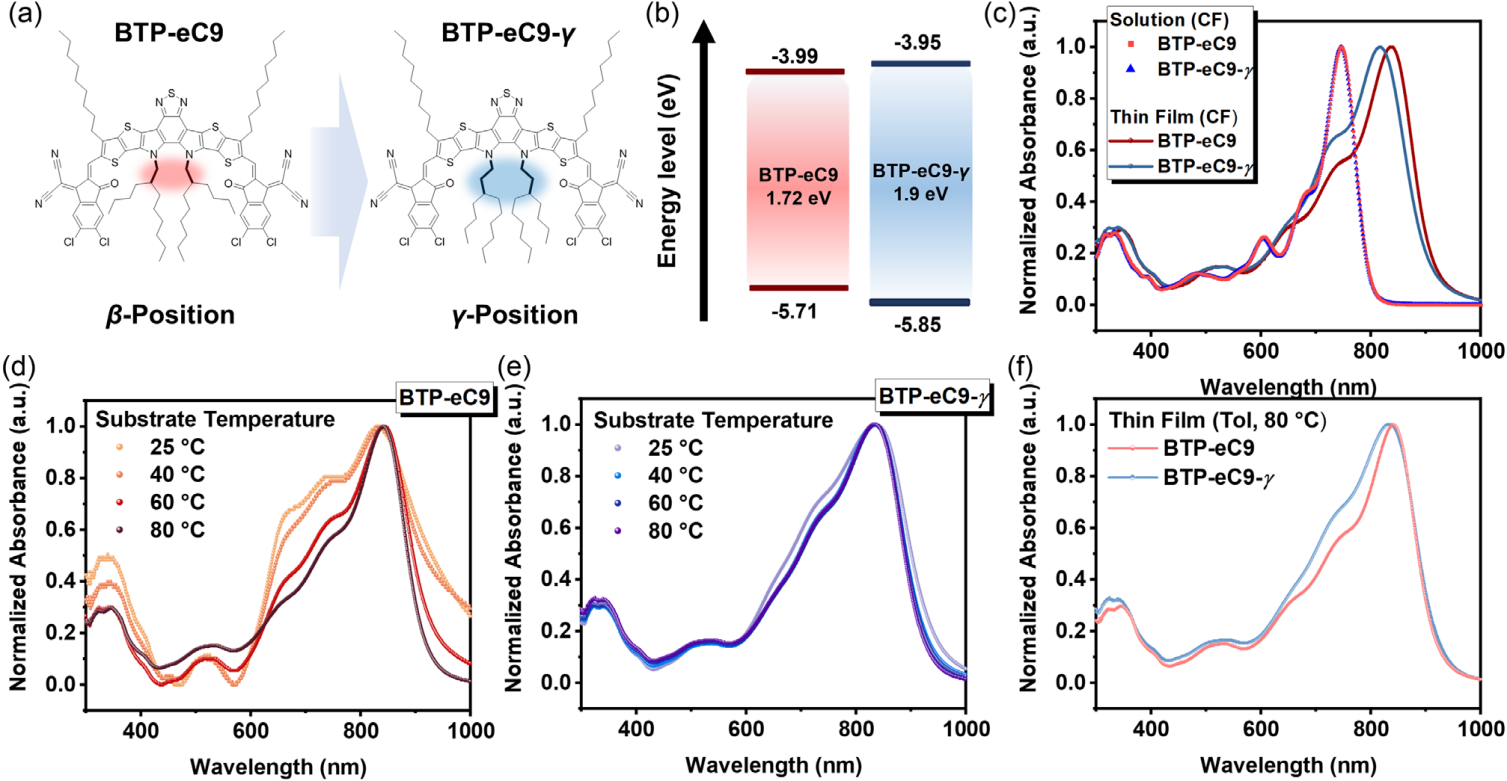
a) Chemical structures and b) energy level diagrams of BTP‐eC9 and BTP‐eC9‐*γ*. c) UV–vis absorption spectra of the neat films in the chloroform solution and thin‐film state. d,e) UV–vis absorption spectra of the blade‐coated neat films processed at different substrate temperatures. f) The difference of *λ*_max_ between the neat films of BTP‐eC9 and BTP‐eC9‐*γ* at 80 °C (toluene).

**Figure** **1**a illustrates the chemical structures of BTP‐eC9 and its variant, BTP‐eC9‐*γ* which features *γ*‐position‐branched inner side chains.^[^ ^11^, ^13^ ^]^ BTP‐eC9‐*γ* was synthesized using procedures similar to those for BTP‐eC9. The details of its synthesis and structural characterization are comprehensively described in the Experimental Section, including synthetic routes, ^1^H and ^13^C nuclear magnetic resonance spectra, and matrix‐assisted laser desorption/ionization‐time of flight mass spectrometry.

As depicted in Figure S1, Supporting Information, thermogravimetric analysis (TGA) indicates that both BTP‐eC9 and BTP‐eC9‐*γ* possess comparable thermal decomposition temperatures (*T*_d_ [5% weight loss] = 307.7 °C for BTP‐eC9 and 307.2 °C for BTP‐eC9‐*γ*). This similarity suggests that the elongation of the branching position in the inner side chains does not significantly impact their thermal stability.^[^ ^14^ ^]^ Furthermore, modifications in the branching position have minimal effect on electron distribution and optimal molecular geometries, as demonstrated by density functional theory calculations at the B3LYP/6‐31G level (refer to Figure S2 and S3, Supporting Information).^[^ ^15^ ^]^ We posit that the theoretical differences in intermolecular packing structures, not addressed herein, may be elucidated through more in‐depth atomic‐level dynamic simulation studies.^[^ ^16^ ^]^

The highest occupied molecular orbital (HOMO) and lowest unoccupied molecular orbital (LUMO) energy levels of both BTP‐eC9 and BTP‐eC9‐*γ* films were determined via cyclic voltammetry (CV), employing Fc/Fc^+^ as an external standard (Figure S4, Supporting Information).^[^ ^17^ ^]^ The calculated HOMO/LUMO values are −5.71/−3.99 eV for BTP‐eC9 and −5.85/−3.95 eV for BTP‐eC9‐*γ* (refer to Figure 1b and **Table** **1**). It is inferred that the extended branching of the inner side chains influences the energy levels, namely, a lower HOMO and slightly higher LUMO, resulting in a larger electrochemical bandgap for BTP‐eC9‐*γ*.

**Table 1:** Optical and electrochemical properties of BTP‐eC9 and BTP‐eC9‐*γ*

NFAs	$\lambda_{\text{max}}^{\text{sol}}$ a) [nm]	$\lambda_{\text{max}}^{\text{CF film}}$ a) [nm]	$\lambda_{\text{max}}^{\text{Tol film}}$ a) [nm]	$\lambda_{\text{onset}}^{\text{film}}$ b) [nm]	*E*_HOMO_ c) [eV]	*E*_LUMO_ c) [eV]	$E_{g}^{\text{CV}}$ c) [eV]
BTP‐eC9	746	837	841	924	−5.71	−3.99	1.72
BTP‐eC9‐*γ*	746	816	833	915	−5.85	−3.95	1.90

a) Obtained from the material solutions in chloroform and corresponding films on glass substrates;

b) Determined from the onset of the UV–vis absorption plots in the films;

c) The HOMO and LUMO energy levels were determined using the equation *E*_HOMO/LUMO_ = −*e*(*E*_ox_/*E*_red_ + 4.8 − *ϕ*_Fc/Fc+_) (eV), where *E*_ox_ and *E*_red_ represent the onsets of oxidation and reduction of the NFAs, respectively, and *ϕ*_Fc/Fc+_ signifies the half‐wave potential of ferrocene versus Ag/Ag^+^, and $E_{g}^{\text{CV}}=E_{\text{LUMO}}\text{ ‐}E_{\text{HOMO}}$(eV).

As presented in Figure 1c and Table 1, the ultraviolet–visible (UV–vis) spectra reveal that both BTP‐eC9 and BTP‐eC9‐*γ* exhibited nearly identical absorption characteristics with dual bands and an identical absorption maximum (*λ*_max_ = 746 nm) in solution. Upon transitioning from solution to film, spin‐casted from chloroform, the *λ*_max_ peak for BTP‐eC9 and BTP‐eC9‐*γ* is red‐shifted to 816 and 837 nm, respectively. Note that the greater degree of red‐shift observed in BTP‐eC9 films, relative to those of BTP‐eC9‐*γ*, indicates increased chain aggregation in the film state.

To delve deeper into the aggregation behavior of both BTP‐eC9 and BTP‐eC9‐*γ* films, we measured their temperature‐dependent UV–vis spectra in the range of 25–80 °C. These films were prepared using blade‐coating with a nonhalogenated toluene solvent, representing the processing conditions used in subsequent OPV device fabrication.^[^ ^18^ ^]^ Notably, BTP‐eC9‐*γ* demonstrates excellent solubility in toluene, while BTP‐eC9 only dissolves effectively in hot toluene. This distinction in solubility (BTP‐eC9 = 0.833 mg mL^−1^ and BTP‐eC9‐*γ* = 15.423 mg mL^−1^ at 25 °C) is evident in Figure S5, Supporting Information, which features a comparative solubility test between the two in toluene. Consequently, this results in less defined spectral‐resolution absorption bands in blade‐coated films processed at lower substrate temperatures (refer to Figure 1d,e). Furthermore, compared to the BTP‐eC9‐*γ* film, the BTP‐eC9 film processed at 80 °C exhibits a slight red‐shift in *λ*_max_, similar to the trend observed in films spin‐casted from chloroform.

### 2.2 Photovoltaic Performances and Charge Transport/Recombination Characteristics

**Figure 2:**
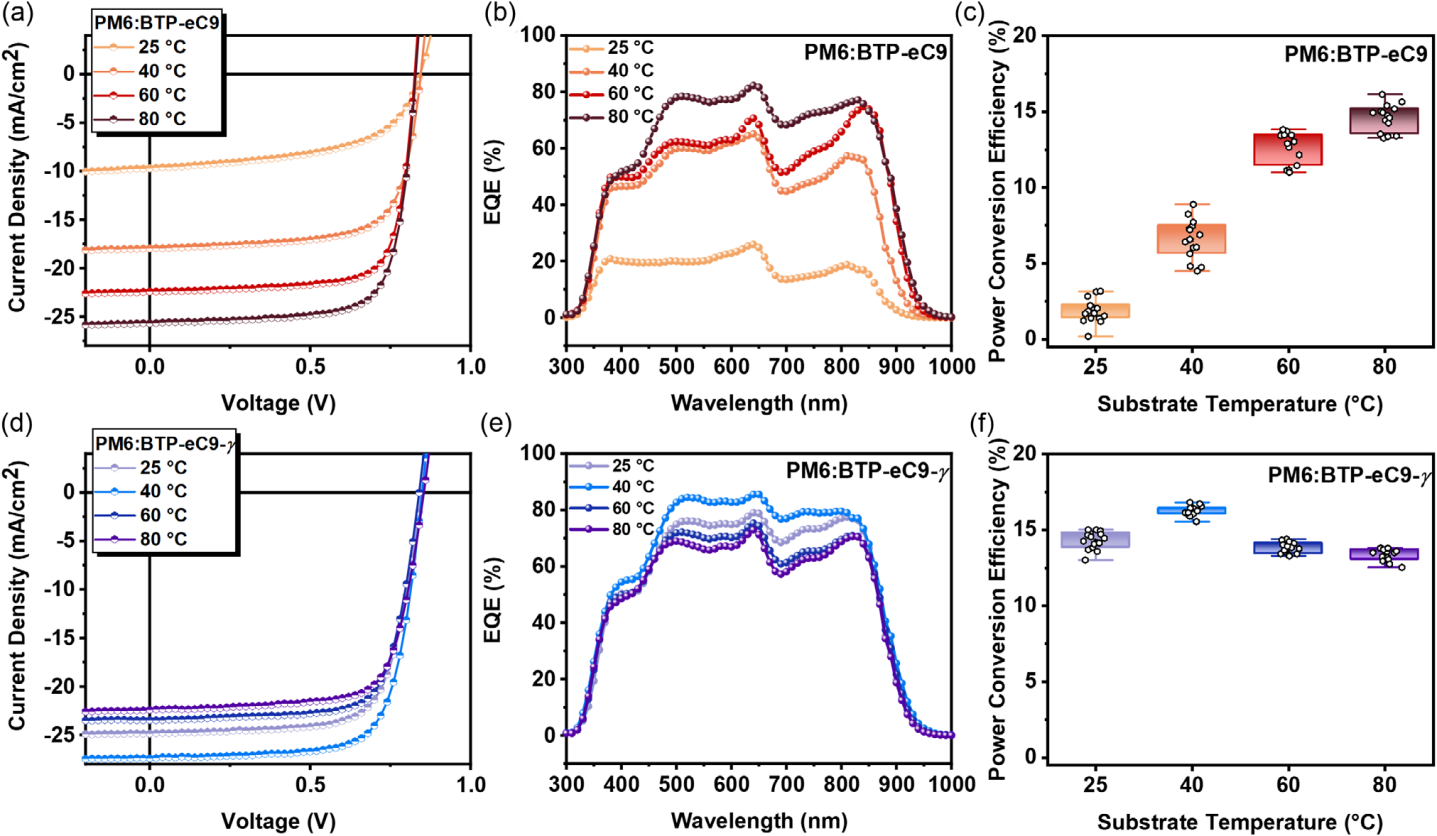
a,d) *J*–*V* curves and b,e) EQE spectra of the OPV devices depending on substrate temperatures. c,f) The distribution histogram of PCEs of the OPV devices depending on substrate temperatures for 15 independently measured devices.

To assess the photovoltaic performance of BTP‐eC9 and BTP‐eC9‐*γ*, we fabricated OPV devices using the blade‐coating technique in a conventional structure: glass/ITO/PEDOT:PSS/active layer/H75/Ag. The H75, previously developed by our group, served as a cathode interlayer.^[^ ^19^ ^]^ The active layer, comprising a blend of PM6 donor polymer and nonfullerene acceptor in toluene solution, was fabricated in an ambient environment. Optimization of the OPV devices, with a blading gap of 100 μm, involved controlling various parameters, including blading speed, donor:acceptor weight ratio, and substrate temperature. The optimization conditions for BTP‐eC9 and BTP‐eC9‐γ are detailed in the Experimental Section. Note that solvent additives (e.g., 1‐chloronaphthelene (CN) and 1,8‐diiodooctane (DIO)) were used for morphology control modulation of each active layer, revealing superior photovoltaic properties in PM6:BTP‐eC9 with DIO and PM6:BTP‐eC9‐*γ* with CN, respectively (refer to Table S8, Supporting Information). A comparative investigation of the best‐performing OPVs was carried out. **Figure** **2**a,d illustrates the representative current density–voltage (*J*–*V*) curves for blade‐coating OPV devices with an active area of 0.04 cm^2^ under simulated AM1.5G one‐sun illumination at 100 mW cm^−2^. The photovoltaic parameters are summarized in **Table** **2**.

**Table 2:** Photovoltaic parameters of optimized devices under illumination of AM 1.5G, 100 mW cm^−2^

Active layer	Substrate temperature [°C]	*V*_OC_ a) [V]	*J*_SC_ a) [mA cm^−2^]	$J_{\text{SC}}^{\text{EQE}}$ [mA cm^−2^]	Fill factor a) [%]	PCEa) [%]
PM6:BTP‐eC9	25	0.817 (0.810 ± 0.007)	7.16 (5.97 ± 1.19)	6.02	46.74 (42.67 ± 4.07)	2.74 (2.15 ± 0.59)
	40	0.818 (0.821 ± 0.003)	17.67 (13.76 ± 3.91)	17.65	52.62 (55.29 ± 2.67)	7.61 (6.25 ± 1.36)
	60	0.827 (0.824 ± 0.003)	21.85 (21.02 ± 0.83)	20.61	73.99 (73.19 ± 0.80)	13.37 (12.81 ± 0.56)
	80	0.836 (0.831 ± 0.005)	25.09 (24.33 ± 0.76)	24.30	76.94 (76.48 ± 0.46)	16.14 (15.46 ± 0.68)
PM6:BTP‐eC9‐*γ*	25	0.847 (0.846 ± 0.001)	24.85 (24.67 ± 0.18)	23.16	71.31 (70.31 ± 1.00)	15.01 (14.32 ± 0.69)
	40	0.860 (0.858 ± 0.002)	25.26 (26.23 ± 0.97)	25.17	75.60 (74.95 ± 0.65)	16.43 (15.97 ± 0.46)
	60	0.857 (0.856 ± 0.001)	23.04 (22.47 ± 0.57)	22.82	73.26 (73.01 ± 0.25)	14.46 (13.81 ± 0.59)
	80	0.854 (0.855 ± 0.001)	22.41 (21.62 ± 0.21)	21.71	72.14 (72.80 ± 0.66)	13.80 (13.37 ± 0.43)

a) The average device values are obtained from 15 cells.

The BTP‐eC9‐based blade‐coating device, with a substrate temperature of 25 °C, exhibited a PCE of 2.74%, a short‐circuit current density (*J*_SC_) of 7.16 mA cm^−2^, an open‐circuit voltage (*V*_OC_) of 0.817 V, and a fill factor of 46.74%. A gradual improvement in OPV performance was observed with increasing substrate temperatures, culminating in a peak PCE of 16.14% at 80 °C.^[10b,10c]^ The lower performance of BTP‐eC9‐based devices at reduced substrate temperatures was attributed to excessive aggregation owing to solubility issues. Conversely, BTP‐eC9‐*γ*‐based blade‐coating devices achieved an optimal PCE of 16.43% at a substrate temperature of 40 °C, with a notably enhanced *V*_OC_ of 0.860 V, primarily due to the down‐shifted HOMO of BTP‐eC9‐*γ*.

Figure 2b,e presents the external quantum efficiency (EQE) spectra for both BTP‐eC9‐based and BTP‐eC9‐*γ*‐based devices across varying substrate temperatures. Figure 2c,f displays the distribution histograms of PCEs for 15 independently measured devices, demonstrating that BTP‐eC9‐*γ*‐based devices exhibit smaller PCE variations compared to BTP‐eC9‐based ones. The calculated *J*_SC_ values from EQE curves closely align with the values of *J*_SC_ obtained from *J*–*V* scans, differing by only 5%, and thus, validating the *J*–*V* measurements.

**Figure 3:**
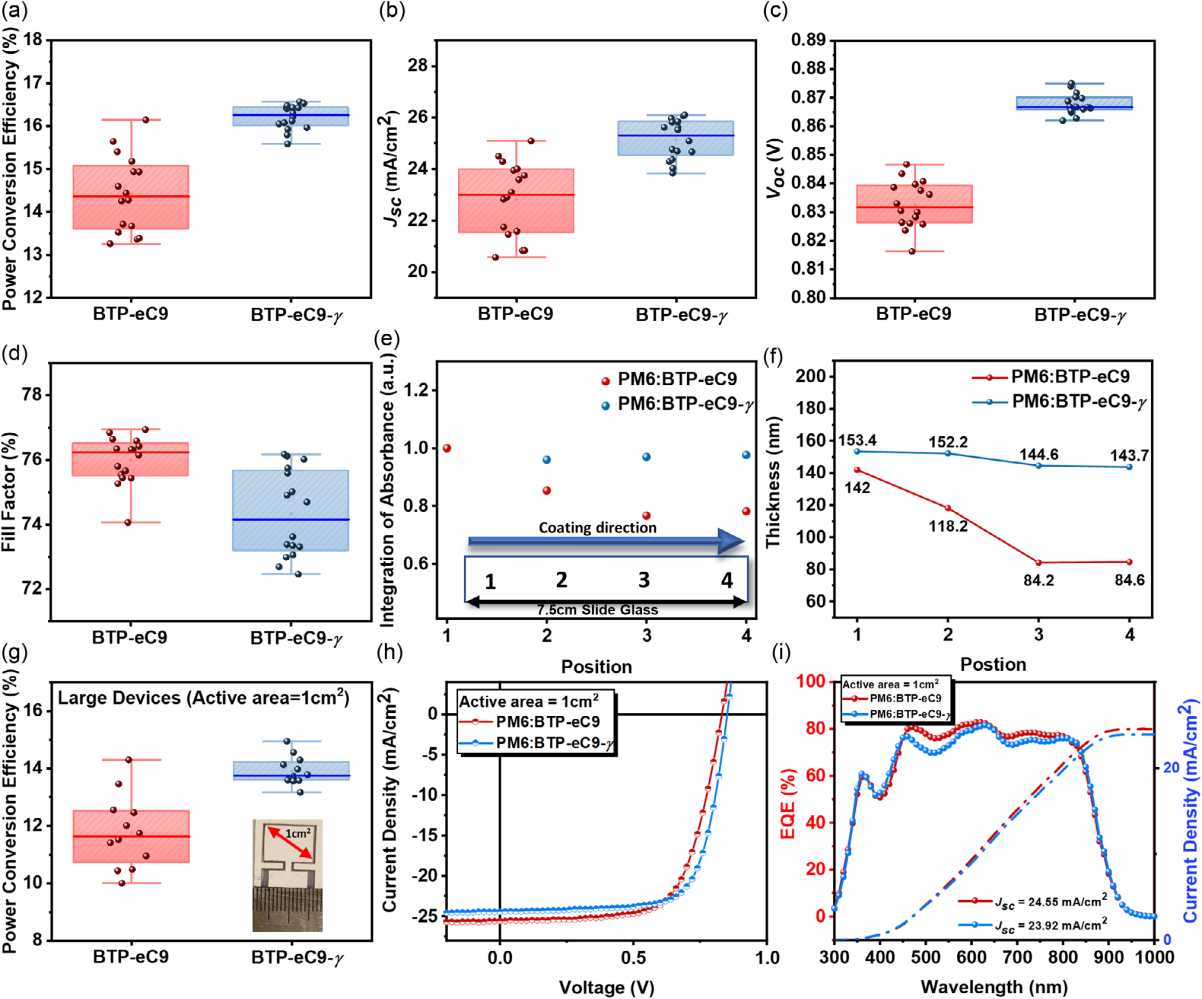
a–d) The distribution histogram of photovoltaic parameters based on PM6:BTP‐eC9‐*γ* and PM6:BTP‐eC9 for 16 independently measured devices. e) Integration of absorbance and f) thickness for each position of the blend films. g) The distribution histogram of PCEs based on PM6:BTP‐eC9‐*γ* and PM6:BTP‐eC9 for 12 independently measured devices. h) The *J*–*V* curves and i) the EQE spectra for the OPV devices.

To further clarify the disparity in PCE variations between BTP‐eC9 and BTP‐eC9‐*γ*‐based devices, we constructed 16 cells for each type under optimal conditions: BTP‐eC9 (80 °C) and BTP‐eC9‐*γ* (at 40 °C). **Figure** **3**a presents statistical data, illustrating significantly reduced PCE variation in BTP‐eC9‐*γ*‐based blade‐coating devices, ranging from 15.58% to 16.42%, compared to those based on BTP‐eC9. These data underscore the superior reproducibility of the BTP‐eC9‐*γ*‐based devices.

To evaluate film uniformity, we prepared large blend films for both BTP‐eC9 at 80 °C and BTP‐eC9‐*γ* at 40 °C on slide glasses (2.0 × 7.5 cm), using identical blade‐coating conditions to those employed in the devices. Figure 3e,f depicts the integrated absorbance bands from 300 to 1000 nm and film thicknesses at four distinct spectral points, positioned at regular intervals along the substrate.^[5b]^ The results indicate that BTP‐eC9‐*γ* films at 40 °C exhibit lower variability in thickness and integrated absorbance compared to BTP‐eC9 films at 80 °C. This finding suggests that blade‐coating at lower substrate temperatures can effectively minimize variations in the evolution kinetics of fluid flow, thereby ensuring consistent film quality and high reproducibility in OPV devices.

Furthermore, we applied the optimized conditions from the small‐scale devices to fabricate large‐area OPV devices over 1.0 cm^2^ for both BTP‐eC9 at 80 °C and BTP‐eC9‐*γ* at 40 °C, using the same conventional device structure. The resultant *J*–*V* curves, including insets of the real device images, and corresponding EQE spectra with calculated *J*_SC_ values are portrayed in Figure 3h,i. Benefiting from homogeneous film production during large‐scale blade‐coating, the BTP‐eC9‐*γ* (40 °C)‐based large‐area devices exhibited higher PCE values (max = 14.95%) with significantly less variation, ranging from 13.16% to 14.95%, as compared to the BTP‐eC9 (80 °C)‐based large‐area devices (PCE = 14.30% with a 4.29% deviation), as highlighted in Figure 3g.^[^ ^20^ ^]^

**Figure 4:**
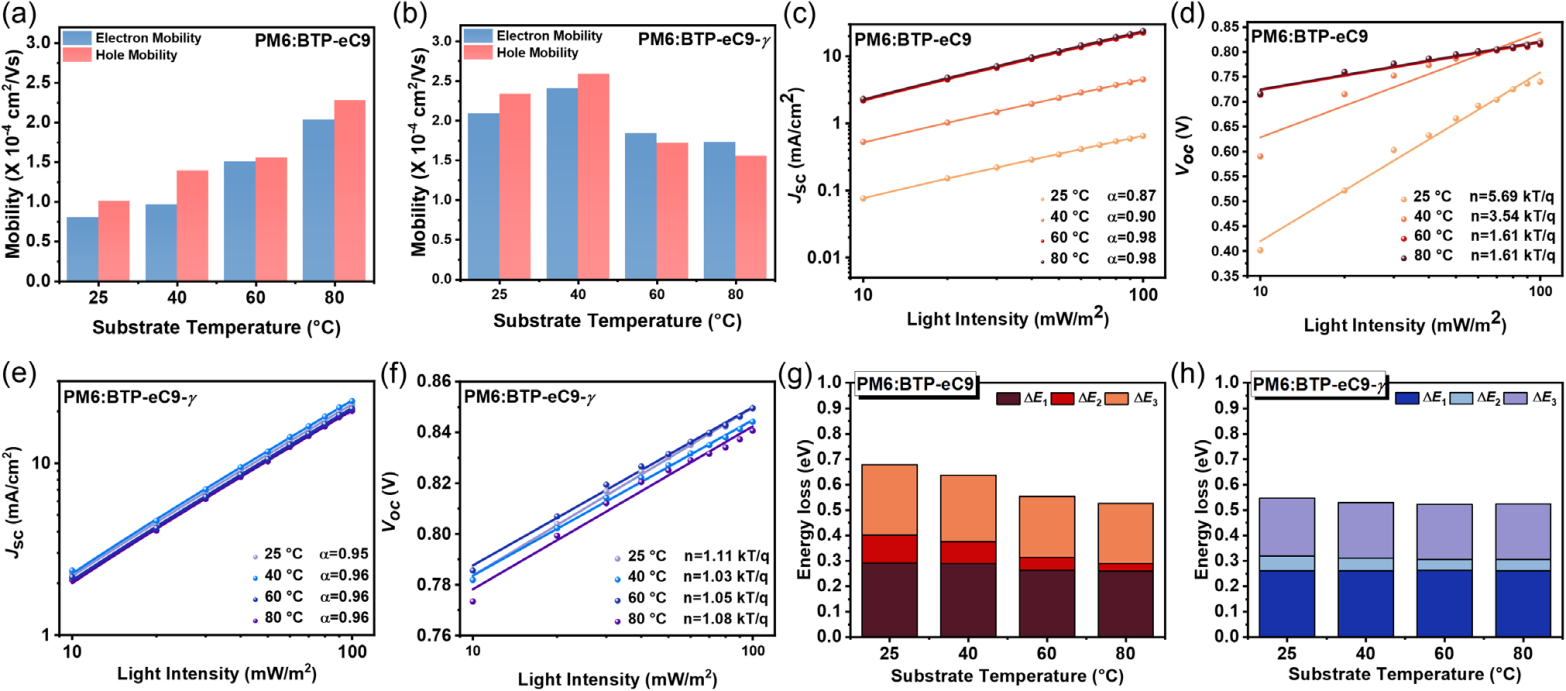
a,b) Hole and electron mobility data of the OPV devices. Light intensity fitting curves of c,e) *J*_SC_ and d,f) *V*_OC_ of the OPV devices. g,h) The total energy loss with each the energy loss terms of the OPV devices.

To discern the underlying reasons for the differing photovoltaic performances of BTP‐eC9 and BTP‐eC9‐*γ*‐based blade‐coating devices processed at various substrate temperatures, we initially evaluated the hole (*μ*_h_) and electron (*μ*_e_) mobilities of the blade‐coating blend films using the space‐charge‐limited current method, as illustrated in Figure S6, Supporting Information. **Figure** **4**a,b demonstrates that BTP‐eC9‐*γ*‐based blend films exhibit overall higher *μ*_h_ and *μ*_e_ values, with less dependence on substrate temperature variations compared to their BTP‐eC9 counterparts. Furthermore, it was observed that both *μ*_h_ and *μ*_e_ values of BTP‐eC9‐based blend films increase as substrate temperatures rise. In contrast, BTP‐eC9‐*γ*‐based blend films processed at lower substrate temperatures (25 and 40 °C) display relatively higher *μ*_h_ and *μ*_e_ values, indicative of the formation of more efficient charge transport networks.^[^ ^21^ ^]^

Furthermore, we investigated the charge recombination behaviors of the devices by measuring the dependence of open‐circuit voltage (*V*_OC_) and short‐circuit current density (*J*_SC_) on light intensity (*P*_light_) ranging from 10 to 100 mW cm^−2^ (Figure 4c–f). The relationships between *P*_light_ versus *J*_SC_ and *P*_light_ versus *V*_OC_ are expressed as *J*_SC_ ∝ *P*_light_^α^ and *V*_OC_ ∝ *n*(*kT*/*q*)ln(*P*_light_), respectively, where *k*, *T*, and *q* represent the Boltzmann constant, temperature (in Kelvin), and charge, respectively. The α value and n slope serve as exponential factors reflecting bimolecular recombination and indicators of monomolecular (trap‐assisted) recombination, respectively.^[^ ^22^ ^]^ For BTP‐eC9‐based blade‐coating devices, substrate temperatures significantly impact both α values (wide ranging from 0.87 to 0.98) and n slopes (wide ranging from 1.61 to 5.69 *kT*/*q*). Specifically, with increasing substrate temperatures, *α* values approach unity and n slopes decrease.

This pattern suggests that carrier recombination in BTP‐eC9‐based devices is highly substrate temperature‐dependent, with reduced recombination being crucial for high‐performing OPV devices, achievable only at elevated substrate temperatures above 60 °C.

Conversely, BTP‐eC9‐*γ*‐based devices show minimal sensitivity to substrate temperatures, exhibiting consistent *α* values ≅ 0.96 and *n* slopes ≅ 1.03–1.11*kT*/*q*. Notably, the best‐performing BTP‐eC9‐*γ*‐based device at a substrate temperature of 40 °C exhibited the lowest *n* slope of 1.03*kT*/*q*, indicating significantly suppressed trap‐assisted charge recombination, which correlates with its improved PCE.^[^ ^23^ ^]^

In addition, we compared the long‐term stability of the unencapsulated BTP‐eC9‐ and BTP‐eC9‐*γ*‐based devices under continuous light (AM 1.5G illumination) (refer to Table S9, Supporting Information and Figure S18, Supporting Information), where an inverted structure of glass/ITO/ZnO/active layer/MoO_3_/Ag was used to eliminate the negative effect induced by high susceptible of organic hole‐ and electron‐transporting layers to chemical and physical degradation upon prolonged light exposure. We found that both BTP‐eC9‐ and BTP‐eC9‐*γ*‐based devices have similar, high‐level retention in PCEs (over 90% of its initial PCE over one week).

### 2.3 Energy Loss

To understand the temperature dependence of the performance of the OPV devices based on BTP‐eC9 and BTP‐eC9‐*γ* with the energy loss, we calculated the total energy loss (*E*_loss_) through EQE electroluminescence (EQE_EL_) measurements (Figure S10, Supporting Information). The *E*_loss_ can be expressed as follows

(1) $$E_{\text{loss}}=\Delta E_{1}+\Delta E_{2}+\Delta E_{3}$$

where Δ*E*_1_ arises from the inevitable radiative recombination due to the Shockley–Queisser limit, Δ*E*_2_ is the additional radiative recombination below the bandgap, and Δ*E*_3_ is the nonradiative recombination. In the case of Δ*E*_3_, we acquired value from the formula ($\Delta E_{3}=-kT\text{ln}\left({\text{EQE}}_{\text{EL}}\right)$).The *E*_loss_ value and related *E*_loss_ terms for each temperature are illustrated and summarized in Figure 4g,h, and Table S3, Supporting Information. In the case of BTP‐eC9 blend systems, Δ*E*_1_ (0.291 and 0.290 eV) and Δ*E*_2_ (0.111 and 0.085 eV) exhibited higher values at lower substrate temperatures under 40 °C. In contrast, the BTP‐eC9‐*γ* blend systems showed almost similar values regardless of the substrate temperatures (0.261–0.262 eV for Δ*E*_1_ and 0.042–0.058 for Δ*E*_2_). At this point, BTP‐eC9‐based devices showed excessive aggregation of BTP‐eC9 at low substrate temperatures owing to lower solubility. Thus, it can lead to the inhibition of charge transport and can induce nonradiative recombination. Especially in the case of Δ*E*_3_ values for the BTP‐eC9‐*γ* blend systems, the highest value was observed at 25 °C (0.226 eV) and it became similar values beyond 40 °C (0.217–0.219 eV). On the contrary, in the BTP‐eC9 blend system, as the substrate temperature was increased, Δ*E*_3_ values were decreased (0.277, 0.262, 0.239, and 0.236 eV for 25, 40, 60, and 80 °C, respectively). The high Δ*E*_3_ value indicates a high nonradiative recombination. BTP‐eC9 exhibited a high nonradiative recombination at low temperatures (under 40 °C), while BTP‐eC9‐*γ* displayed lower nonradiative recombination under similar conditions. Additionally, the *E*_g_ values were higher at lower substrate temperatures in both blend systems, exhibiting 1.496 eV for BTP‐eC9 and 1.402 eV for BTP‐eC9‐*γ*. Consequently, BTP‐eC9‐*γ* demonstrated a higher *E*_g_ and lower total energy loss at lower temperatures, elucidating why BTP‐eC9‐*γ* performed the highest *V*_OC_ at 40 °C. Therefore, based on the aforementioned data, BTP‐eC9‐*γ* blend systems exhibited the optimal substrate temperatures at 40 °C, while in BTP‐eC9 blend systems, the optimal performance was shown at the high substrate temperature (80 °C). This performance enhancement is potentially attributable to the suitable blend morphology, as described in the subsequent morphology study.

### 2.4 Blend Film Morphology

**Figure 5:**
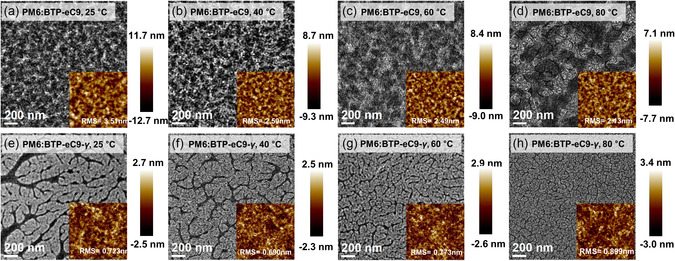
a–h) TEM and AFM height images of the blend films depending on substrate temperatures.

To investigate the temperature‐dependent blend morphology, we conducted atomic force microscopy (AFM), transmission electron microscopy (TEM), surface energy (*γ*) measurements, and grazing‐incidence wide‐angle X‐ray scattering (GIWAXS) on the blade‐coating blend films. AFM images revealed that BTP‐eC9‐*γ*‐based blend films, especially those processed at low substrate temperatures, have smoother surfaces with significantly lower root–mean–square roughness values (ranging from 0.690 to 0.899 nm) compared to BTP‐eC9‐based films. As depicted in **Figure** **5**, TEM imaging indicates indistinct phase‐separation morphologies in BTP‐eC9‐based films, with moderate phase‐separation only observed in the film processed at 80 °C.^[^ ^24^ ^]^

In contrast, BTP‐eC9‐*γ*‐based films exhibit multilength fibrillar‐like phase separations. The domain size and quality of these films diminish at higher substrate temperatures. Specifically, the film processed at 40 °C demonstrates uniform phase distribution with optimal domain sizes, whereas the films processed at lower (25 °C) and higher (60–80 °C) substrate temperatures display oversized domains and lower‐quality phase separations.^[^ ^24^, ^25^ ^]^

To assess miscibility between donor and acceptor through the Flory–Huggins interaction ($\chi_{\text{da}}=K{\left[\sqrt{\gamma_{\text{donor}}}\;–\;\sqrt{\gamma_{\text{acceptor}}}\right]}^{2}$), we measured the γ of their respective neat films processed at different substrate temperatures (refer to Figure S7 and S8, Supporting Information). Note that the degree of accuracy of *γ* values of BTP‐eC9 at low substrate temperatures may be compromised owing to its poor solubility as discussed. As collected in Table S1, Supporting Information, the *χ*_da_ values are evaluated to be 0.675 (25 °C), 1.520 (40 °C), 0.620 (60 °C), and 0.280 (80 °C) for PM6:BTP‐eC9 blends and 0.203 (25 °C), 0.058 (40 °C), 0.108 (60 °C), and 0.198 (80 °C) for PM6:BTP‐eC9‐*γ* blends, respectively.

The lower *χ*_da_ values suggest the increased miscibility between the two components. Based on this analysis, the higher miscibility in PM6:BTP‐eC9‐*γ* blends, especially at 40 °C with the lowest *χ*_da_ value, promotes the formation of a completely mixed morphology with high domain purity.^[^ ^26^ ^]^ This finding is consistent with the morphological characteristics observed in the AFM and TEM images. Such enhanced miscibility is a key factor contributing to the improved photovoltaic performance of the BTP‐eC9‐*γ*‐based device at 40 °C.

Additionally, we measured the contact angles of PM6:BTP‐eC9 and PM6:BTP‐eC9‐*γ* blend solutions on the PEDOT:PSS‐coated ITO glass. It is found that relative to PM6:BTP‐eC9 one (15.2°), PM6:BTP‐eC9‐*γ* has a smaller contact angle (10.5°), indicating its better surface wettability and/or contact interface characteristics on the PEDOT:PSS‐coated ITO glass.

**Figure 6:**
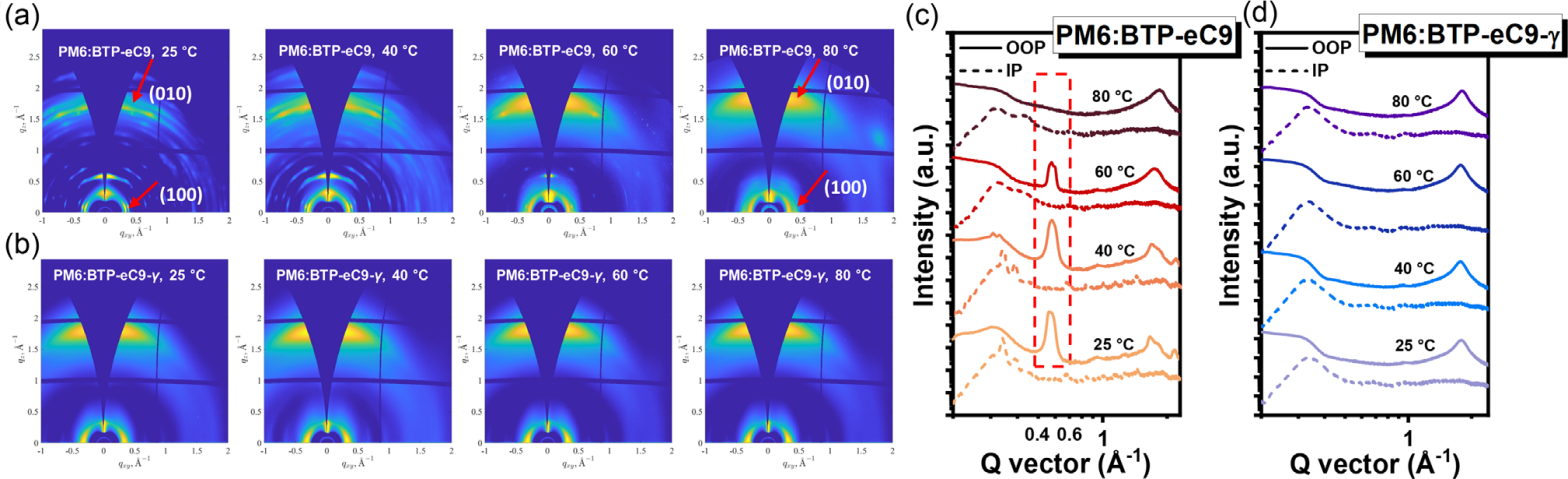
a,b) 2D GIWAXS diffraction patterns of the blend films depending on substrate temperatures. c,d) The corresponding line‐cut profiles for the blend films.

The GIWAXS patterns and corresponding line‐cut profiles of the blend films are presented in **Figure** **6**, with detailed crystallographic parameters summarized in Table S4 and S5, Supporting Information. All blends exhibit a pronounced (010) diffraction peak in the out‐of‐plane (OOP) direction and a (100) diffraction peak in the in‐plane (IP) direction, indicating a predominant face‐on orientation relative to the substrate.^[^ ^27^ ^]^ We observed that BTP‐eC9‐based blends at substrate temperatures ranging from 25 °C to 60 °C display numerous diffraction spots and a marked diffraction peak at *q* = 0.58 Å^−1^ in the OOP direction, originating from BTP‐eC9 molecules.^[10b]^

In contrast, for BTP‐eC9‐based blends at 80 °C, only two significant diffraction peaks are evident, namely, (010) OOP and (100) IP. These findings suggest aggressive aggregation characteristics owing to strong crystalline properties and low solubility of BTP‐eC9 at lower temperatures. Such excessive aggregations are further corroborated by notable variations in UV−Vis spectra and the presence of visible particles with a feature size of ≈100 μm in optical microscopy (OM) images of BTP‐eC9 blend films processed at low temperatures (refer to Figure S11 and S12, Supporting Information). These factors are primarily responsible for the diminished device performance of BTP‐eC9‐based devices at low substrate temperatures.

Intriguingly, all BTP‐eC9‐*γ* blends display only distinct (010) OOP and (100) IP peaks without any additional scattering signals, indicative of the formation of homogeneous mixed‐phase networks, regardless of substrate temperature. Consistently, almost identical UV−Vis spectra and prominent OM images are observed for all BTP‐eC9‐*γ* blends (Figure S11 and S12, Supporting Information), further emphasizing the uniformity in their structural formation.

## 3 Conclusion

In conclusion, we have successfully synthesized BTP‐eC9‐*γ* by extending the branching position of inner side chains from the β‐position to *γ*‐position within BTP‐eC9. This modification was aimed at enhancing the efficacy of blade‐coating OPV device manufacturing using nonhalogenated toluene solvent. The BTP‐eC9‐*γ* film exhibited a larger electrochemical bandgap and blue‐shifted absorbance relative to BTP‐eC9. Additionally, this alteration in the branching position contributed to the suppression of severe chain aggregation in the film state, thereby improving solubility, and processability at lower temperatures.

Consequently, BTP‐eC9‐*γ*‐based blade‐coating devices, combining low substrate temperature of 40 °C with toluene, achieved remarkable PCEs of 16.43% for a small size of 0.04 cm^2^ and 14.95% for a larger area of 1.0 cm^2^. In contrast, high‐performance BTP‐eC9‐based blade‐coating devices were only attainable at high substrate temperature of 80 °C. Notably, the BTP‐eC9‐*γ*‐based devices processed at 40 °C demonstrated exceptional reproducibility and high film quality, attributed to minimal variation in the morphology evolution kinetics of fluid flow. The present findings underscore the potential of strategically modifying the branching position within the molecular structures to develop efficient, highly reproducible blade‐coating OPV devices—a crucial advancement for the industrialization of OPV technology.

## 4 Experimental Section

4.1

4.1.1

##### Materials and Characterization

All starting materials were purchased from Sigma‐Aldrich, Acros, or TCI and used without further purification. All solvents are ACS and anhydrous grade unless otherwise noted.

^1^H nuclear magnetic resonance (NMR) and ^13^C NMR spectra were recorded on a Bruker Avance III HD 400 MHz (Bruker) spectrophotometer using CDCl3 as a solvent containing tetramethylsilane as the internal standard. TGA was measured using Q500 (TA instruments, USA) with a scan rate of 10 °C min^−1^ in nitrogen flow. UV–vis spectra were obtained by a Shimadzu UV‐2550 spectrometer. CV measurements were measured with an AMETEK Versa STAT 3 with a three‐electrode cell system in a nitrogen‐bubbled 0.1 m tetra‐n‐butylammonium hexafluorophosphate (n‐Bu_4_NPF_6_) in acetonitrile (CH_3_CN) solution at a scan rate of 100 mV^−1^ s^−1^ at room temperature. An Ag/Ag^+^ electrode, platinum wire, and glassy carbon electrode were performed as the reference electrode, counter electrode, and working electrode, respectively. The matrix assisted laser desorption/ionization time‐of‐flight mass spectrometry mass spectrometry experiments were performed on an autoflex max instrument (Bruker). Density functional theory calculations were performed using the Gaussian 16 package with the nonlocal hybrid Becke three‐parameter Lee–Yang–Parr (B3LYP) function and the 6–31G basis set to elucidate the HOMO and LUMO levels after optimizing the structural configurations for BTP‐eC9 and BTP‐eC9‐*γ* using the same method.

##### Device Fabrication and Measurement

The OPV devices were fabricated with the conventional configuration of glass/ITO/PEDOT:PSS/active layer/H75/Ag and the inverted configuration of Glass/ITO/ZnO/Active layer/MoO_3_/Ag. The patterned ITO–coated glass substrate was cleaned using detergent, DI water, acetone, and isopropanol for 15 min for each step, subsequently. After the ITO glass substrates were treated with UV–ozone for 15 min, PEDOT:PSS (Bayer Baytron 4083) was spin‐coated at 4000 rpm onto the ITO substrate, followed by annealing at 150 °C for 20 min in air. The PM6:BTP‐eC9‐*γ* (1:1.2 weight ratio) were dissolved in toluene at a concentration of 15 mg mL^−1^ with 0.5 vol% CN additive while 0.5 vol% DIO additive was employed in PM6:BTP‐eC9 (1:1.2 weight ratio), followed by a hotplate heated at 80 °C for 2 h for both cases. The active layers in solutions were blade‐coated onto the PEDOT:PSS‐coated ITO glass (blading speed 15 mm s^−1^). The temperatures of the blend solutions were the same as those of the substrate. Then, a methanol solution of H75 (2.0 mg mL^−1^) was deposited onto the active layer via spin–coating at 3000 rpm for 30 s. Finally, 100 nm Ag was thermally evaporated under vacuum (<3.0 × 10–6 Pa). The J–V characteristics were measured on a Keysight B2900 sourcemeter under the illumination of an AM 1.5G solar simulator with an intensity of 100 mW cm^−2^. The EQE measurements were conducted using a Model QE‐R3011 (Enli Technology) in ambient air. The hole and electron mobilities were measured using the space charge limited current (SCLC) method. Device structures are ITO/PEDOT:PSS/photoactive layer/MoO_3_/Ag for hole‐only devices and ITO/ZnO/photoactive layer/H75/Ag for electron‐only devices. The SCLC mobilities were calculated using the Mott–Gurney equation

(2) $$J=\frac{9}{8}\varepsilon_{0}\varepsilon_{r}\mu \left(\frac{V^{2}}{L^{3}}\right)$$

where *ε*_0_ is the permittivity of empty space, *ε*_r_ is the relative dielectric constant of the organic semiconductor, *μ* is the mobility of zero‐field, *V* is the applied voltage across the device, and *L* is the thickness of the active layer. The EQE measurements were conducted using a Model QE‐R3011 (Enli Technology) in ambient air.

##### Morphology Characterization

The investigation of thin‐film morphology was performed using AFM, TEM, contact angles, and GIWAXS measurements. The samples were prepared by blade‐coating on the glass substrate (for AFM and TEM) or silicon substrate (for GIWAXS) using the solution of NFAs (2 mg mL^−1^ in Toluene) for the neat film and the optimized device fabrication condition for the blend films. AFM and TEM were measured via a Hitachi AFM5100N in the tapping mode and Tecnai G2 F20 X‐Twin transmission electron microscope equipped with an energy‐dispersive X‐ray analysis at an acceleration voltage of 200 kV, respectively. The contact angle test was performed on a SmartDrop (Femtofab Co., Ltd., Korea), and the surface energy of the acceptors and PM6 was evaluated by measuring the contact angles using two different solvents: DI water and ethylene glycol on each neat film and calculated via the Owens−Wendt model. The corresponding blend miscibility between the donor and acceptor was estimated by the Flory−Huggins interaction parameter (*χ*) calculated by the following equation

(3) $$\chi =K{\left(\sqrt{\gamma_{\text{donor}}}–\sqrt{\gamma_{\text{acceptor}}}\right)}^{2}$$

where *K* is a positive proportional constant, and *γ*_abnor_ and *γ*_acceptor_ represent the surface energies of the donor and acceptor materials, respectively.

The solution wettability test was conducted under the same conditions as the fabrication of the device, with each blend solution dropped onto a PEDOT:PSS film, and then contact angles were measured. GIWAXS was carried out at the PLS‐II 3C U‐SAXS beamline of the Pohang Accelerator Laboratory in Korea.

## Conflict of Interest

The authors declare no conflict of interest.

## Supporting information

Supplementary Material

## Data Availability

The data that support the findings of this study are available from the corresponding author upon reasonable request.
