# Supplementary material for: Low‐Temperature Processed Efficient and Reproducible Blade‐Coating Organic Photovoltaic Devices with γ‐Position Branched Inner Side Chains‐Containing Nonfullerene Acceptor
Source: Small Sci. 2024 Apr 15;4(7):2400034. doi: 10.1002/smsc.202400034 (PMC11935161; doi:10.1002/smsc.202400034)
Supplement: Supplementary file 1 — Supplementary Material [file SMSC-4-2400034-s001.pdf]

Supporting Information

Low-Temperature Processed Efficient and Reproducible Blade-Coating Organic Photovoltaic Devices with γ-Position Branched Inner Side Chains-Containing Nonfullerene Acceptor

Donghoo Won[1†], So-Huei Kang[1], Jaeyeong Park[1], Jeewon Park[1], Wonjun Kim[1], Thi Le Huyen Mai[1], Seunglok Lee[1], and Changduk Yang[1,2]*

[1]Department of Energy Engineering, School of Energy and Chemical Engineering, Perovtronics Research Center, Low Dimensional Carbon Materials Center, Ulsan National Institute of Science and Technology (UNIST), 50 UNIST-gil, Ulju-gun, Ulsan 44919, South Korea.

[2]Graduate School of Carbon Neutrality, Ulsan National Institute of Science and Technology (UNIST), 50 UNIST-gil, Ulju-gun, Ulsan 44919, South Korea.

*Corresponding author. Email: yang@unist.ac.kr

Synthetic Procedure

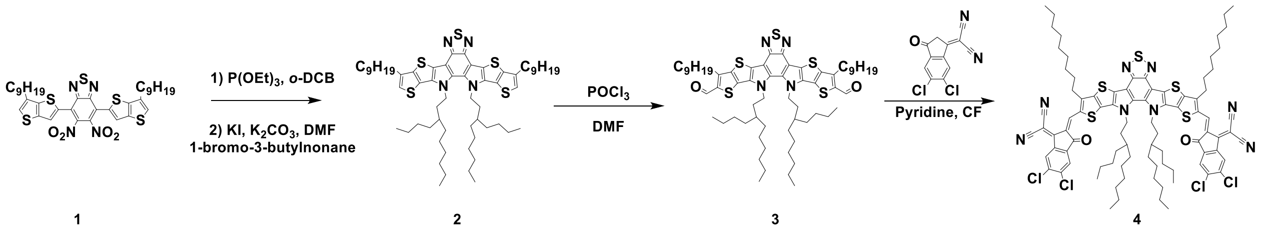

Scheme S1. Synthetic routes of BTP-eC9-*γ*

12,13-Bis(3-butylnonyl)-3,9-dinonyl-12,13-dihydro-[1,2,5]thiadiazolo[3,4-e]thieno[2'',3'':4',5']thieno[2',3':4,5]pyrrolo[3,2-g]thieno[2',3':4,5]thieno[3,2-b]indole (2)

Compound 1 (1 g, 1.32 mmol) and triethyl phosphite (15 ml) were dissolved in the *o*-dichlorobenzene (*o*-DCB, 5 ml) into the two-neck flask and purged with argon. After being heated at 180 °C overnight, the aqueous phase was extracted with dichloromethane (DCM) and the organic layer was dried over $MgSO_4$ and filtered. After solvent removal, the red crude residue remained in the flask. Then, DMF (20 ml), potassium hydroxide (1.83 g, 13.24 mmol), potassium iodide (0.22 g, 1.32 mmol), and 1-bromo-3-butylnonane (1.65 g, 6.62 mmol) were added to the flask and purged with argon for 20 minutes. The mixture was refluxed at 80 °C for overnight. After solvent removal from the filtrate, the residue was subjected to extraction with ethyl acetate and water. The organic layers were combined, dried over $MgSO_4$, filtered, and further purified through column chromatography on silica gel, utilizing a dichloromethane/hexane (1/1, v/v) eluent. This process yielded a red solid (0.74 g, 53% yield). ^{1}H NMR (400 MHz, $CDCl_3$, δ): 7.11 (s, 2H), 4.79 (t, J = 7.5 Hz, 4H), 2.91 (t, J = 7.7 Hz, 4H), 1.95 (p, J = 7.5 Hz, 4H), 1.78 (d, J = 7.4 Hz, 4H), 1.50 (dt, J = 18.3, 7.0 Hz, 3H), 1.38 (s, 21H), 1.25 – 1.11 (m, 9H), 1.11 – 0.81 (m, 28H), 0.78 – 0.66 (m, 6H).

12,13-bis(3-butylnonyl)-3,9-dinonyl-12,13-dihydro-[1,2,5]thiadiazolo[3,4-e]thieno[2'',3'':4',5']thieno[2',3':4,5]pyrrolo[3,2-g]thieno[2',3':4,5]thieno[3,2-b]indole-2,10-dicarbaldehyde (3)

Phosphorus oxychloride (1.46 mL, 15.72 mmol) was dissolved in DMF (5 ml) in a two-neck flask and purged with argon at 0 °C. After heating and stirring at room temperature for 1 hour. At the same time, compound 2 (0.7g, 0.66 mmol) was dissolved in dichloroethane (DCE, 20 ml) in another two-neck flask, also purged with argon. Subsequently, a solution of $POCl_3$ was added to the flask containing compound 2. The mixture was then heated and stirred at 90 °C for overnight. After cooling down, saturated Na_2CO_3 (Dissolved in water) solution was added to the reactant and the mixture was stirred for 1 hour. The crude was subjected to extraction with dichloromethane and water. The organic layers were combined, dried over $MgSO_4$, filtered, and further purified through column chromatography on silica gel, utilizing a dichloromethane/hexane (7/3, v/v) eluent. This process yielded an orange solid (0.77 g, 86% yield). ^{1}H NMR (400 MHz, $CDCl_3$, δ): 10.14 (s, 2H), 4.76 (t, J = 7.3 Hz, 4H), 3.20 (t, J = 7.7 Hz, 4H), 1.92 (p, J = 7.6 Hz, 4H), 1.69 (q, J = 7.1 Hz, 4H), 1.52 – 1.34 (m, 8H), 1.28 (s, 20H), 1.05 (s, 11H), 0.91 (dt, J = 22.6, 7.2 Hz, 10H), 0.73 (t, J = 7.2 Hz, 9H), 0.61 (td, J = 7.2, 1.1 Hz, 6H).

2,2'-((2Z,2'Z)-((12,13-bis(3-butylnonyl)-3,9-dinonyl-12,13-dihydro-[1,2,5]thiadiazolo[3,4-e]thieno[2'',3'':4',5']thieno[2',3':4,5]pyrrolo[3,2-g]thieno[2',3':4,5]thieno[3,2-b]indole-2,10-diyl)bis(methaneylylidene))bis(5,6-dichloro-3-oxo-2,3-dihydro-1H-indene-2,1-diylidene))dimalononitrile (4, BTP-eC9-*γ*)

Compound 3 (0.77 g, 0.69 mmol) and 2-(5, 6-dichloro-3-oxo-2,3-dihydro-1H-inden-1-ylidene)malononitrile (1.09 g, 4.16 mmol) were dissolved in chloroform in a two-neck flask and purged with argon. Subsequently, pyridine was added to the flask, and the mixture was heated to 80 °C while being stirred overnight. After cooling to room temperature, the product was precipitated by adding methanol (200 ml) and then filtered. The crude further purified through column chromatography on silica gel, utilizing a dichloromethane/hexane (4/6, v/v) eluent. This process yielded a dark blue solid (0.7 g, 63%). ^{1}H NMR (400 MHz, $CDCl_3$, δ):, 8.84 (s, 2H), 8.69 (s, 2H), 7.85 (s, 2H), 4.73 (d, J = 8.3 Hz, 4H), 2.99 (s, 4H), 1.89 (s, 4H), 1.80 – 1.66 (m, 4H), 1.45 (d, J = 7.8 Hz, 3H), 1.27 (d, J = 12.5 Hz, 30H), 1.19 – 0.92 (m, 21H), 0.92 – 0.82 (m, 5H), 0.73 (td, J = 6.9, 3.2 Hz, 13H). ^{13}C NMR (100 MHz, $CDCl_3$, δ): 185.86, 153.77, 147.01, 145.20, 139.33, 138.98, 138.52, 135.82, 113.66, 77.24, 68.98, 33.75, 31.99, 31.90, 30.96, 29.83, 29.47, 29.41, 29.32, 29.04, 26.93, 23.07, 22.74, 22.71, 14.16, 14.13, 14.11.; HRMS(MALDI-TOF): calcd for $C_{88}H_{98}Cl_4N_8O_2S_5$, 1601.91. Found: 1601.49

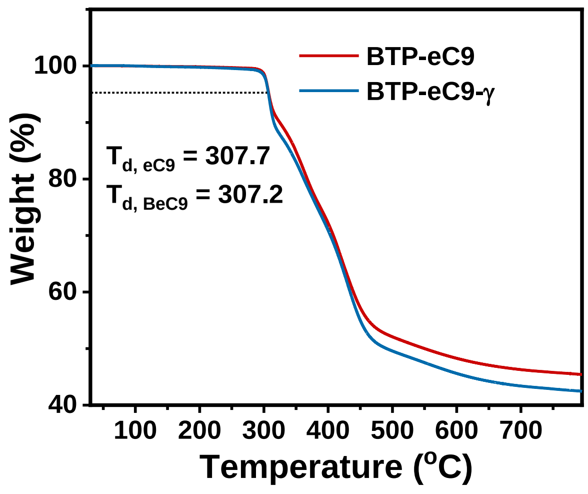

Figure S1. TGA curves of BTP-eC9 and BTP-eC9-*γ*.

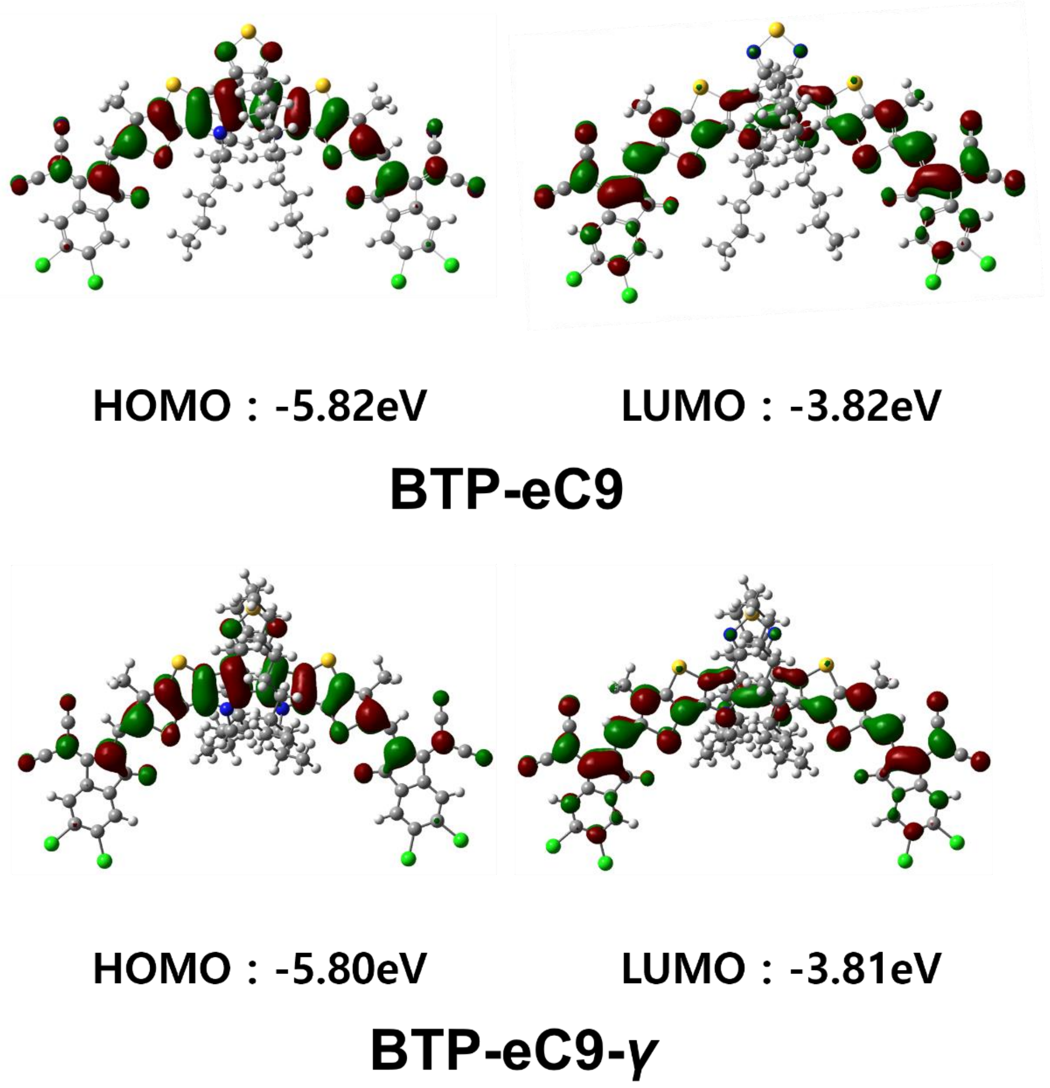

Figure S2. Simulated HOMO and LUMO distributions by DFT calculation.

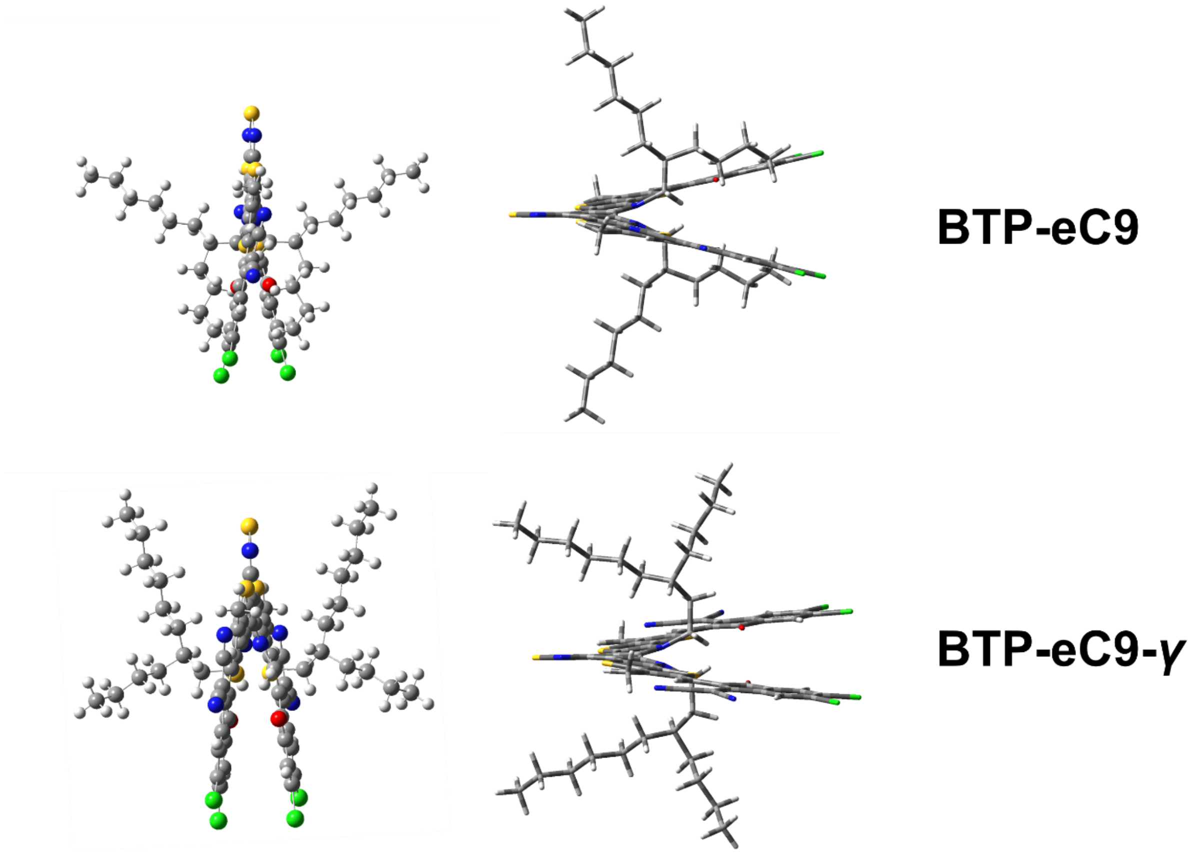

Figure S3. Simulated structure by DFT calculation.

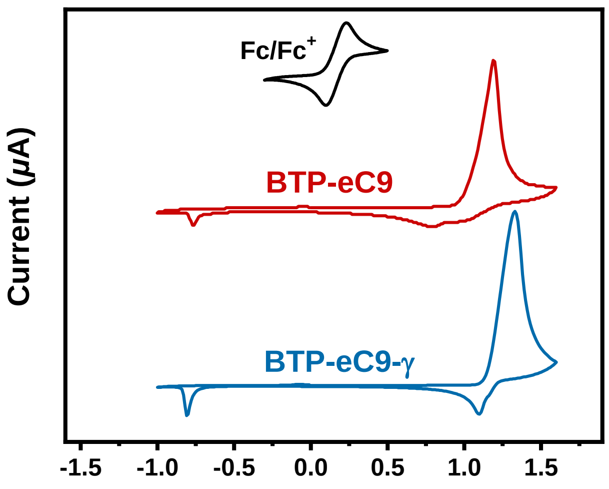

Figure S4. Cyclic voltammetry curve of PM6:BTP-eC9-γ and PM6:BTP-eC9.

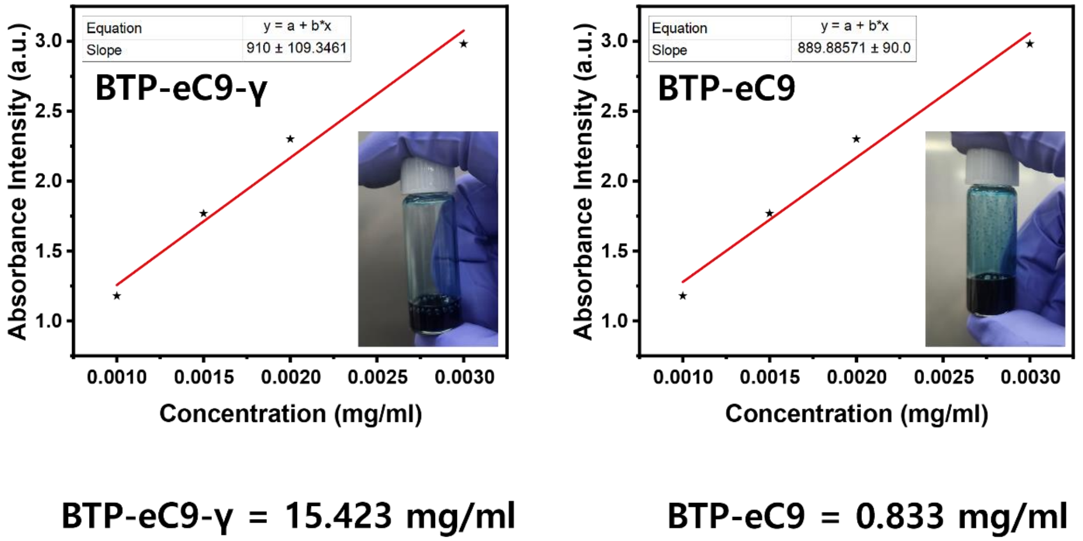

Figure S5. Solubility test of BTP-eC9-*γ* and BTP-eC9 in Toluene at room temperature.

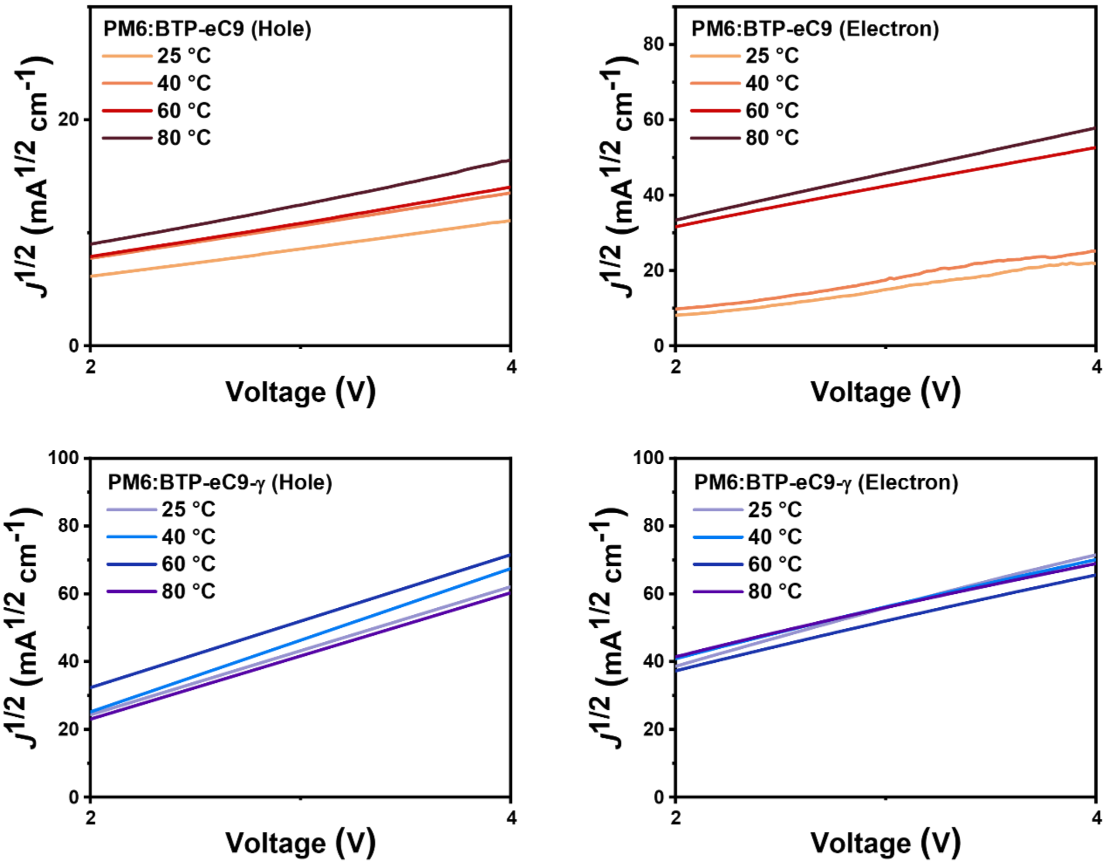

Figure S6. SCLC fitting curve of PM6:BTP-eC9-*γ* and PM6:BTP-eC9.

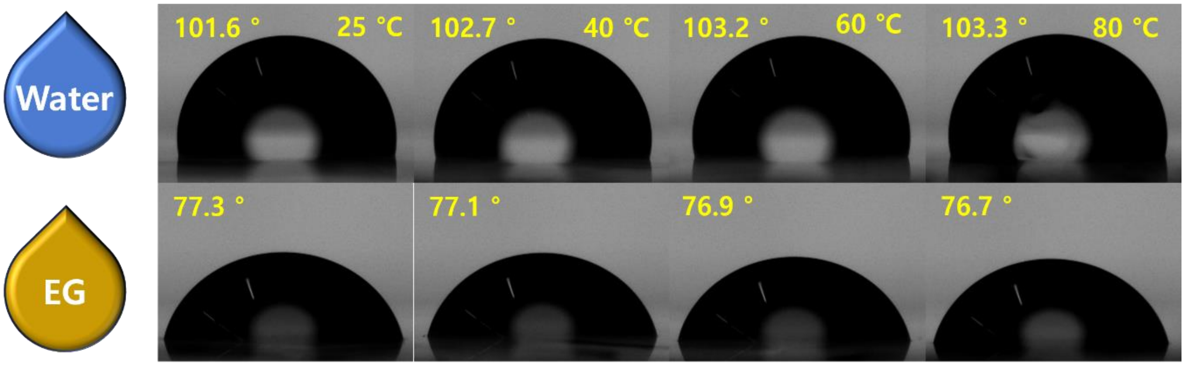

Figure S7. Contact angle images of PM6 neat films.

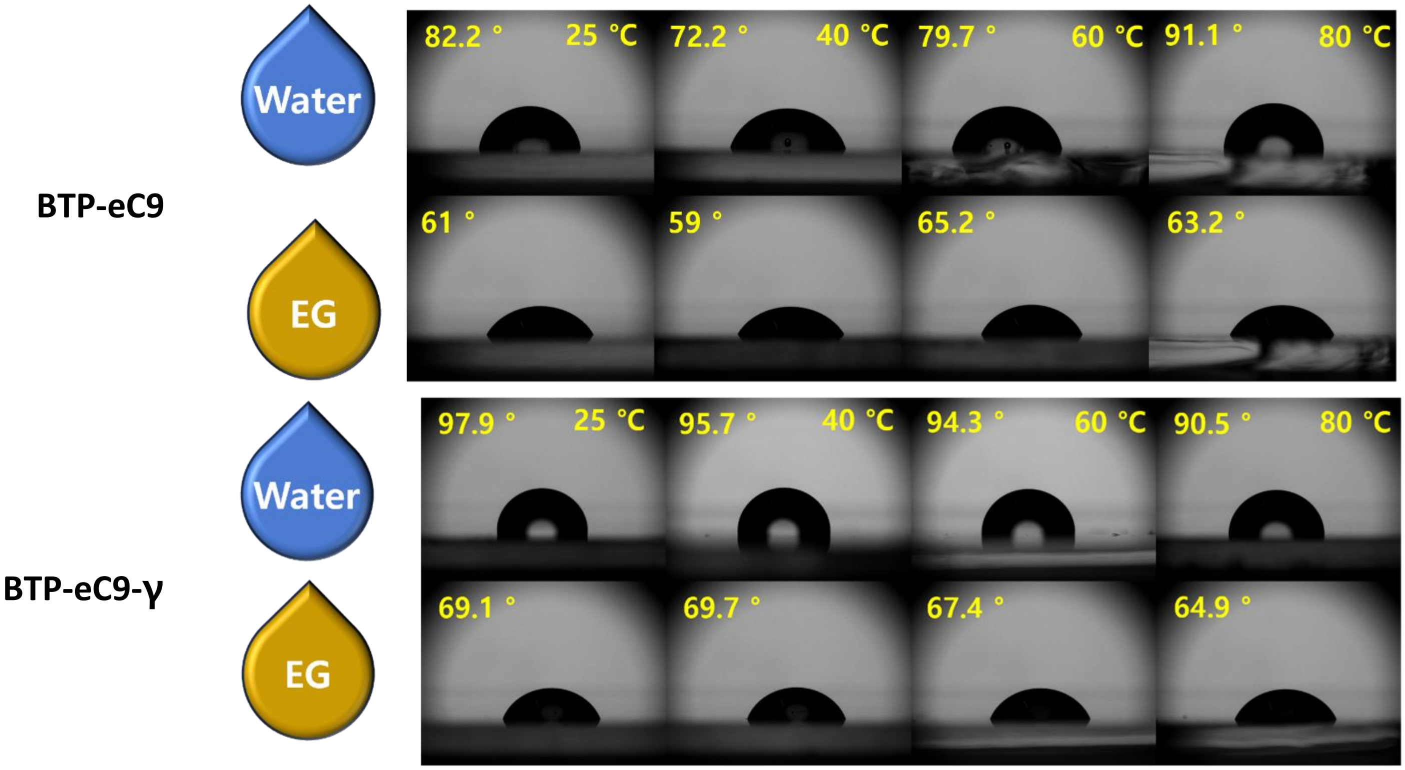

Figure S8. Contact angle images of BTP-eC9 and BTP-eC9-*γ* neat films.

Table S1. Contact angle parameters of the neat films of PM6, BTP-eC9 and BTP-eC9-*γ*.

	Substrate Temperature [°C]	Water [°]	EG [°]	γ^d [mN/m]	γ^p [mN/m]	γ [mN/m]
**PM6**	25	101.6	77.3	14.9	6.4	21.3
	40	102.7	77.1	16.6	5.3	21.9
	60	103.2	76.9	17.6	4.7	22.3
	80	103.3	76.7	18.0	4.5	22.5

	Substrate Temperature [°C]	Water [°]	EG [°]	γd [mN/m]	γp [mN/m]	γ [mN/m]
**BTP-eC9**	25	82.2	61	12.9	16.7	29.6
	40	72.2	59	9.7	25.6	35.3
	60	79.7	65.2	9.3	21.1	30.4
	80	91.1	63.2	17.9	9.5	27.4

	Substrate Temperature [°C]	Water [°]	EG [°]	γd [mN/m]	γp [mN/m]	γ [mN/m]
**BTP-eC9-γ**	25	97.9	69.1	19.7	6.0	25.7
	40	95.7	69.7	16.3	8.2	24.5
	60	94.3	67.4	17.1	8.4	25.6
	80	90.5	64.9	15.8	10.8	26.5

Table S2. Flory-Huggins interaction parameters.

Substrate Temperature [°C]	χ (BTP-eC9-γ)	χ (BTP-eC9)
25	0.203	0.675
40	0.058	1.520
60	0.108	0.620
80	0.198	0.280

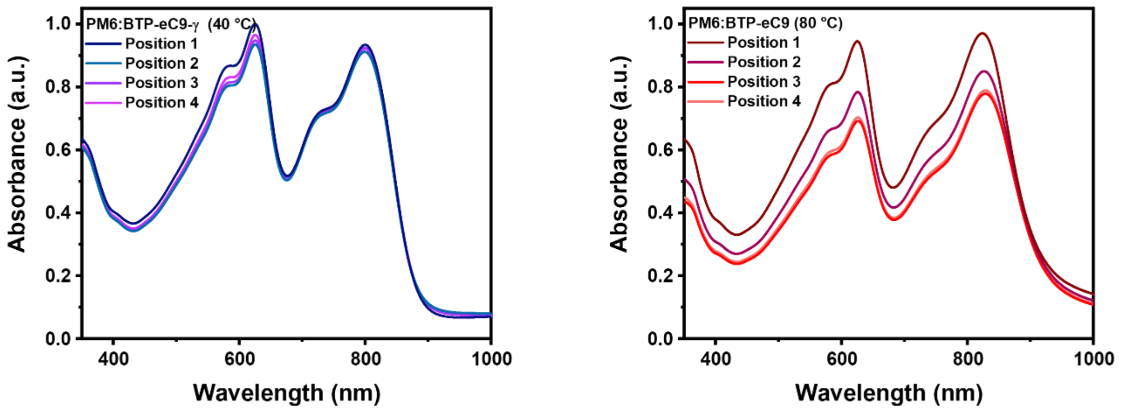

Figure S9. UV absorbance data of PM6:BTP-eC9 and PM6:BTP-eC9-γ depending on substrate temperatures for each position.

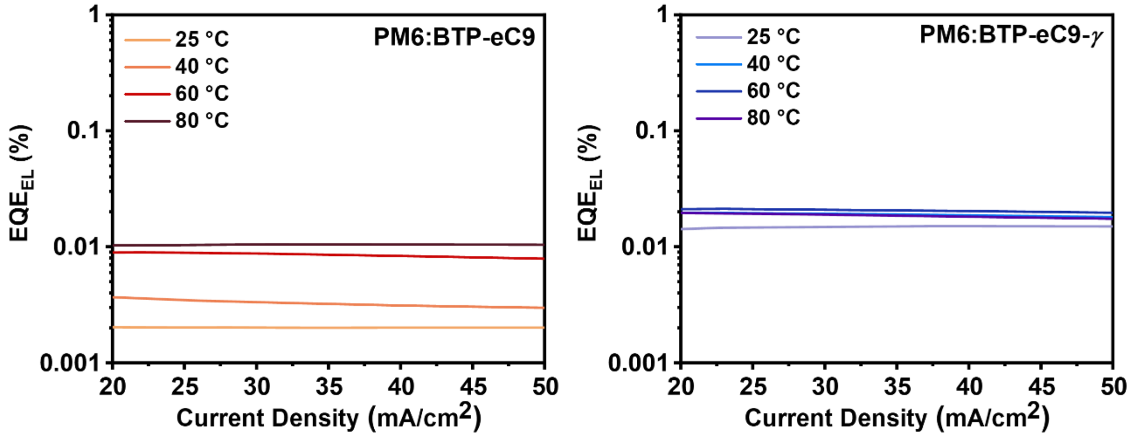

Figure S10. EQE_{EL} data for PM6:BTP-eC9 and PM6:BTP-eC9-γ.

Table S3. The total energy loss terms of PM6:BTP-eC9-γ and PM6:BTP-eC9.

Active layer	Substrate Temperature [°C]	V_{OC} [V]	E_{loss} [eV]	ΔE_1 [eV]	ΔE_2 [eV]	ΔE_3 [eV]	E_g [eV]
PM6:BTP-eC9	25	0.817	0.679	0.291	0.111	0.277	1.496
	40	0.818	0.637	0.290	0.085	0.262	1.455
	60	0.827	0.554	0.263	0.052	0.239	1.381
	80	0.836	0.526	0.259	0.031	0.236	1.359
PM6:BTP-eC9-γ	25	0.847	0.546	0.261	0.058	0.226	1.402
	40	0.860	0.529	0.262	0.048	0.219	1.391
	60	0.857	0.522	0.262	0.042	0.217	1.383
	80	0.854	0.524	0.262	0.042	0.219	1.381

Table S4. GIWAXS parameters of the PM6:BTP-eC9 blend films.

Processing temperature (PM6:BTP-eC9)		25 °C	40 °C	60 °C	80 °C
Fom q_z profile IP (100)	q (Å^{-1})	0.339	0.342	0.320	0.318
	d-spacing (Å)	18.536	18.372	19.635	19.730
	FWHM (Å^{-1})	0.014	0.015	0.052	0.060
	Coherence length (Å)	409.660	384.337	109.316	94.647

Processing temperature (PM6:BTP-eC9)		25 °C	40 °C	60 °C	80 °C
Fom q_z profile OOP (010)	q (Å^{-1})	1.722	1.741	1.772	1.831
	d-spacing (Å)	3.649	3.609	3.546	3.432
	FWHM (Å^{-1})	0.211	0.218	0.219	0.223
	Coherence length (Å)	27.063	26.247	26.135	25.660

Table S5. GIWAXS parameters of the PM6:BTP-eC9-γ blend films.

Processing temperature (PM6:BTP-eC9-γ)		25 °C	40 °C	60 °C	80 °C
Fom q_z profile IP (100)	q (Å^{-1})	0.339	0.335	0.337	0.335
	d-spacing (Å)	18.526	18.730	18.661	18.731
	FWHM (Å^{-1})	0.076	0.069	0.066	0.064
	Coherence length (Å)	74.185	81.966	85.769	88.701
Processing temperature (PM6:BTP-eC9-γ)		25 °C	40 °C	60 °C	80 °C
Fom q_z profile OOP (010)	q (Å^{-1})	1.789	1.770	1.775	1.802
	d-spacing (Å)	3.512	3.550	3.540	3.487
	FWHM (Å^{-1})	0.1997	0.205	0.208	0.210
	Coherence length (Å)	28.667	27.884	27.491	27.290

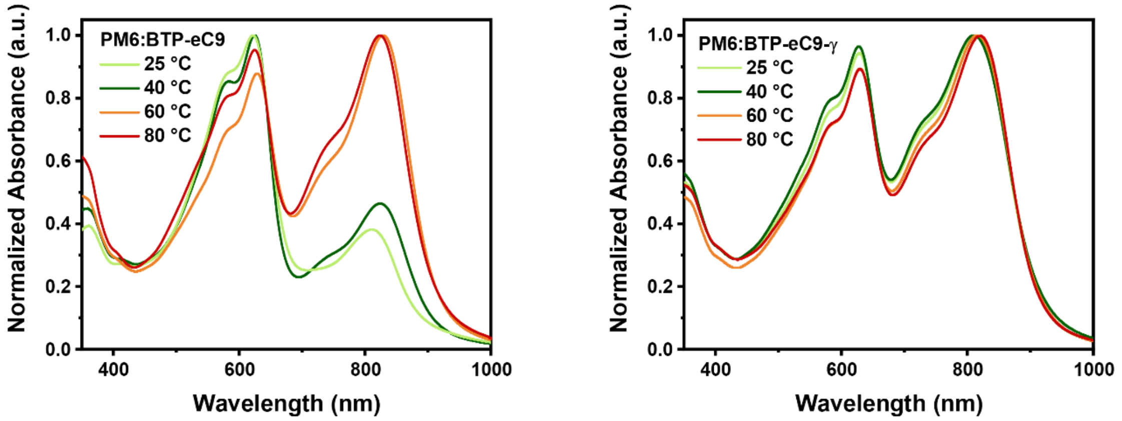

Figure S11. UV absorbance data of PM6:BTP-eC9 and PM6:BTP-eC9-γ depending on substrate temperatures.

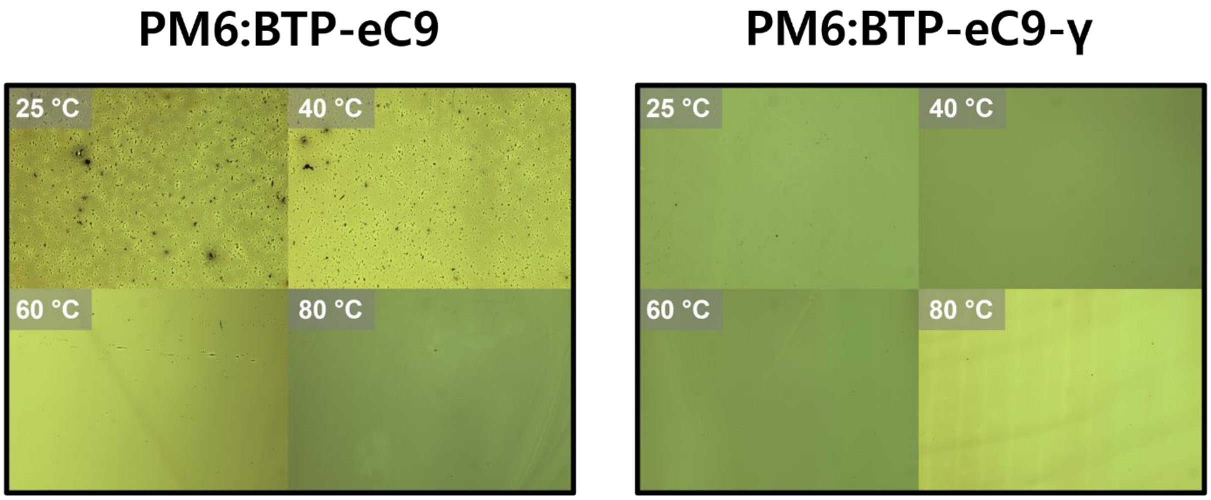

Figure S12. Image of optical microscopy of PM6:BTP-eC9 and PM6:BTP-eC9-*γ* depending on substrate temperatures.

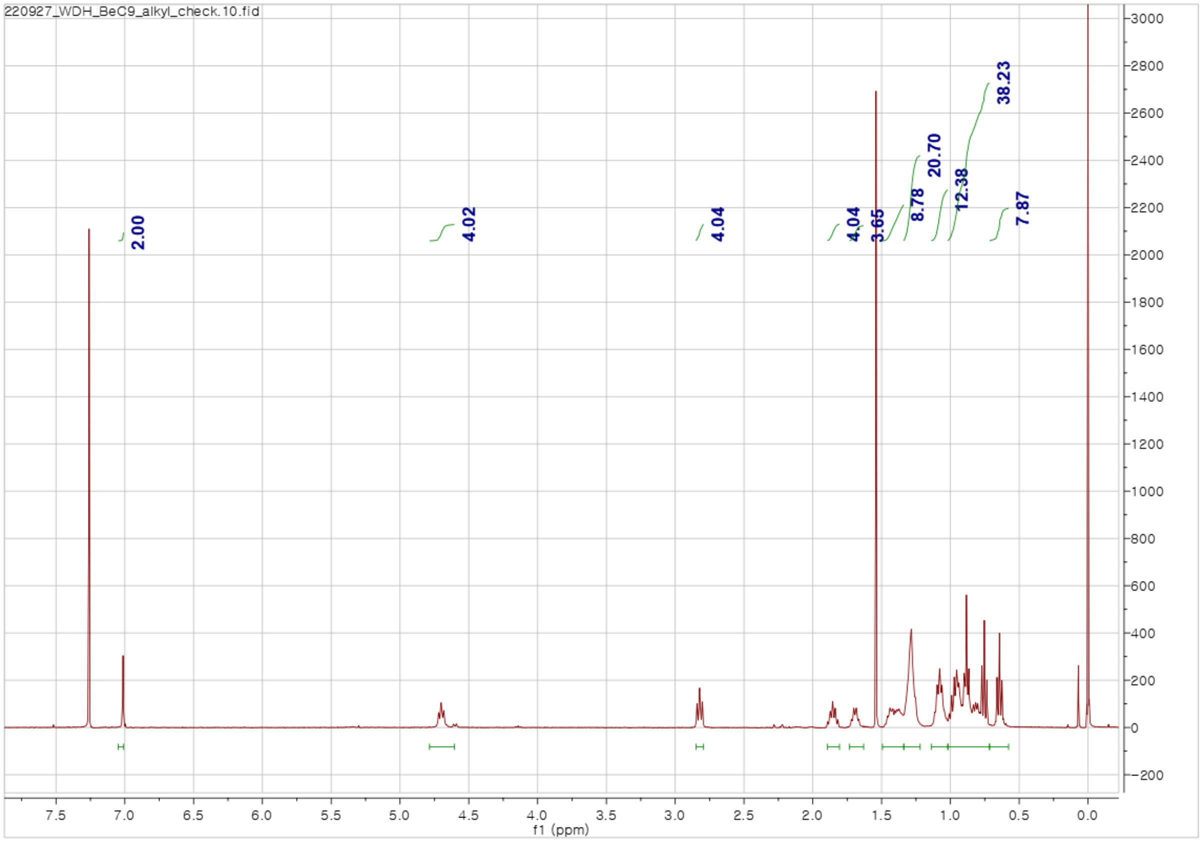

Figure S13. ^{1}H-NMR spectrum of compound **2**.

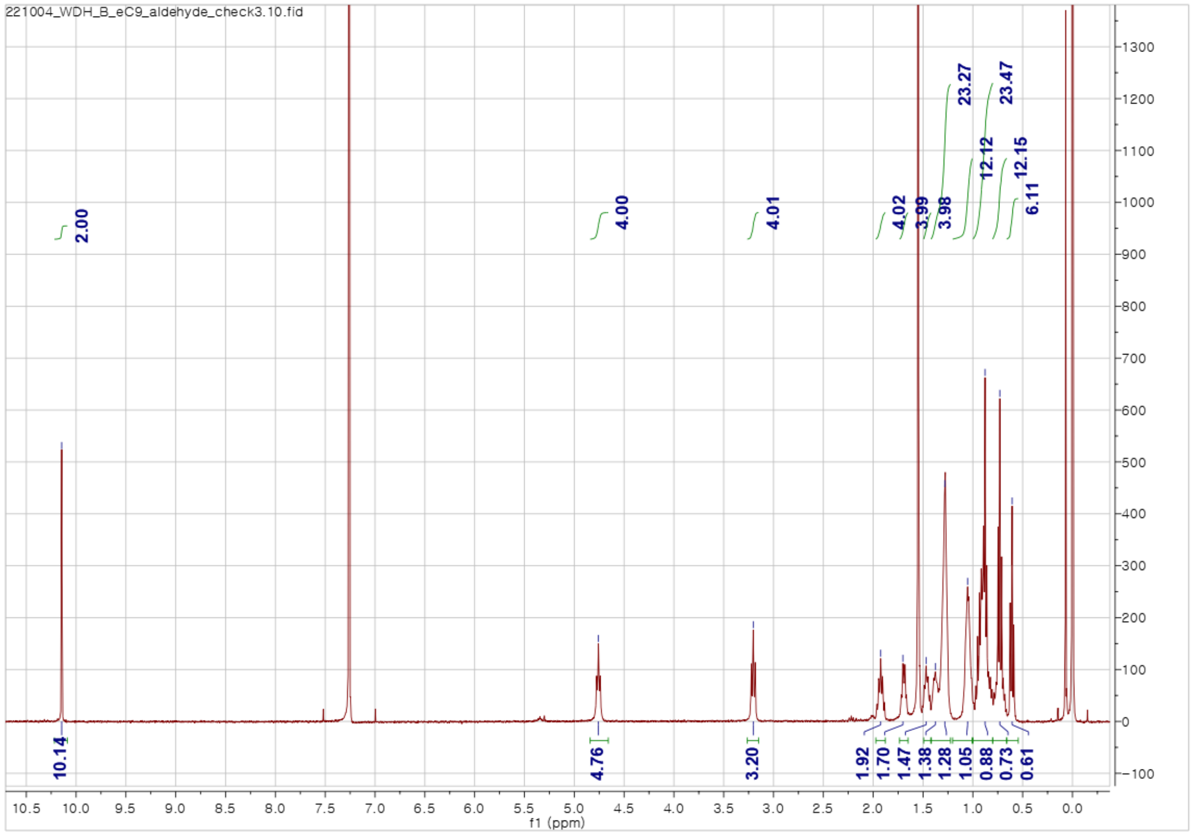

Figure S14. ^{1}H-NMR spectrum of compound **3**.

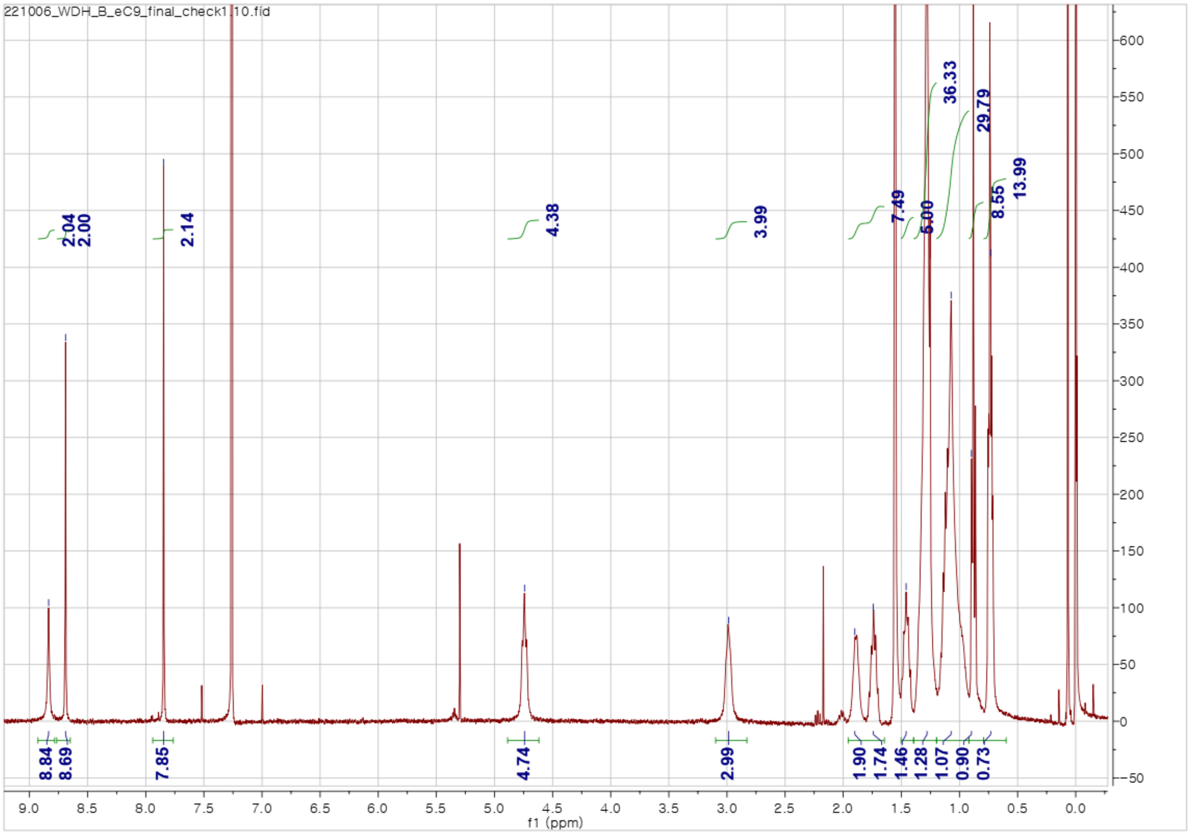

Figure S15. ^{1}H-NMR spectrum of compound **4** (**BTP-eC9-*γ***).

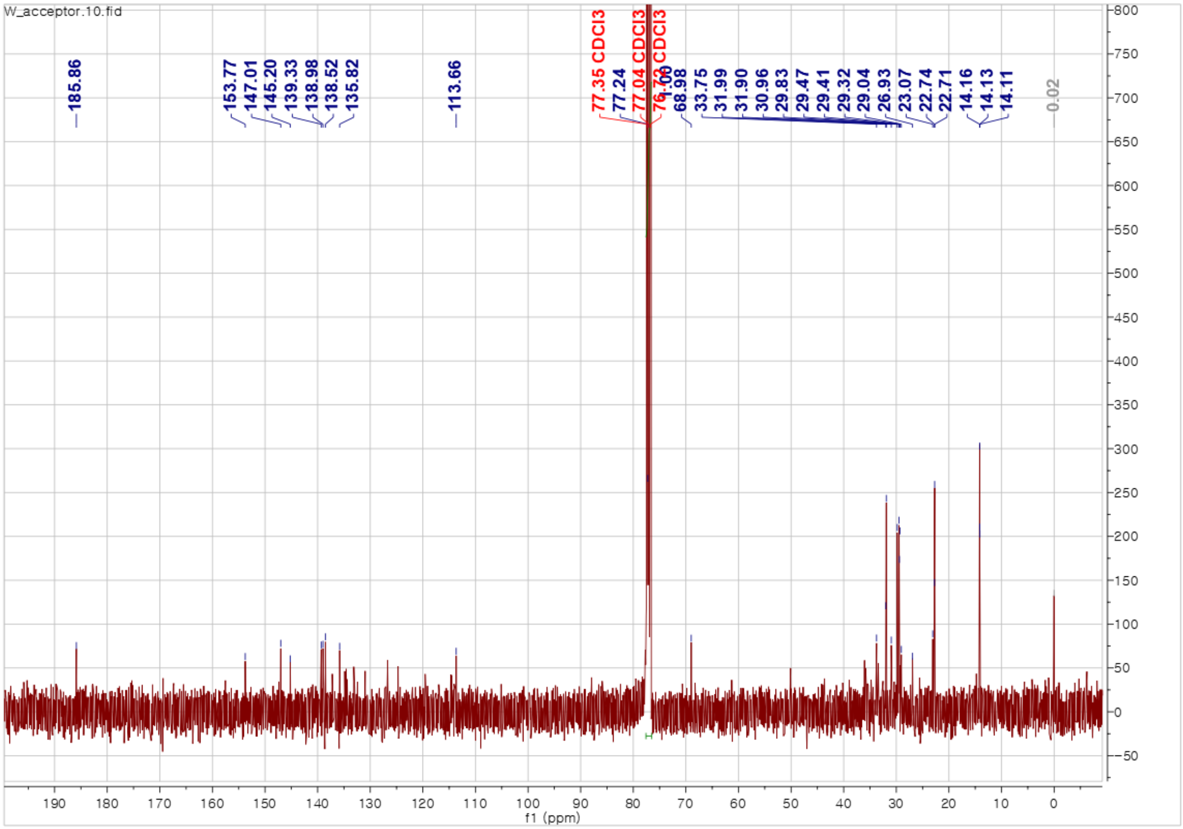

Figure S16. ^{13}C-NMR spectrum of compound **4** (**BTP-eC9-*γ***).

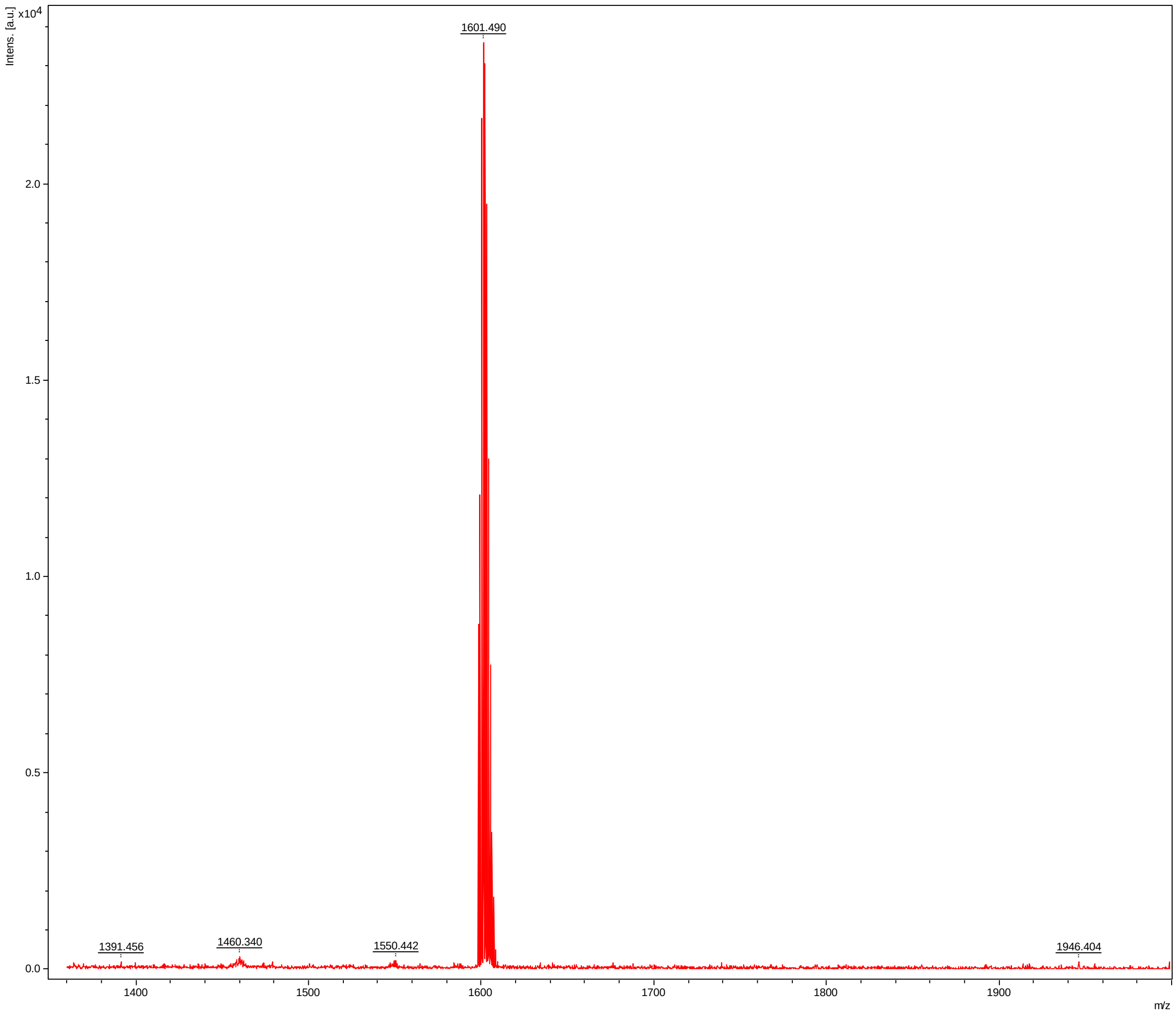

Figure S17. MALDI-TOF spectrum of compound **4(BTP-eC9-*γ*)**.

Table S6. SCLC fitting data of **PM6:BTP-eC9-*γ*** and **PM6:BTP-eC9**

NFAs	Substrate Temperature [°C]	μ_h [× 10^{-4} cm^2 V^{-1} s^{-1}]	μ_e [× 10^{-4} cm^2 V^{-1} s^{-1}]	μ_h / μ_e
BTP-eC9	25	1.01	0.8	1.26
	40	1.39	0.96	1.45
	60	1.55	1.5	1.03
	80	2.28	2.03	1.12
BTP-eC9-γ	25	2.34	2.09	1.12
	40	2.59	2.41	1.07
	60	1.72	1.84	0.93
	80	1.55	1.73	0.90

Table S7. Photovoltaic parameters of **PM6:BTP-eC9-*γ*** and **PM6:BTP-eC9 (1 cm^2)**

Active layer	Substrate Temperature [°C]	Active area [cm^2]	V_{oc} [a)] [V]	J_{sc} [a)] [mA/cm^2]	J_{sc}^{EQE} [mA/cm^2]	Fill Factor [a)] [%]	PCE [a)] [%]
PM6:BTP-eC9	80	1.01	0.831 (0.827 ± 0.004)	25.59 (23.23 ± 2.36)	24.55	71.48 (65.47 ± 6.01)	14.30 (12.61 ± 1.69)
PM6:BTP-eC9-γ	40	1.01	0.851 (0.846 ± 0.005)	24.44 (22.96 ± 1.48)	23.92	71.88 (66.82 ± 5.06)	14.95 (13.97 ± 0.98)

[a)] the average device values are obtained from 12 cells.

Table S8. Optimization for photovoltaic parameters of PM6:BTP-eC9-*γ* and PM6:BTP-eC9 (0.04 cm^2)

Active layer	Additive	Substrate Temperature [°C]	V_{OC}[V]	J_{SC} [mA/cm^2]	Fill Factor [%]	PCE [%]
PM6:BTP-eC9	CN (0.5 vol %)	25	0.723	1.00	35.17	0.26
		40	0.781	5.21	39.23	1.60
		60	0.821	18.92	64.19	9.97
		80	0.854	25.06	74.21	15.89
	DIO (0.5 vol %)	25	0.817	7.16	46.74	2.74
		40	0.818	17.67	52.62	7.61
		60	0.827	21.85	73.99	13.37
		80	0.836	25.09	76.94	16.14
PM6:BTP-eC9-γ	CN (0.5 vol %)	25	0.847	24.85	71.31	15.01
		40	0.860	25.26	75.60	16.43
		60	0.857	23.04	73.26	14.46
		80	0.854	22.41	72.14	13.80
	DIO (0.5 vol %)	25	0.818	25.88	65.68	13.91
		40	0.831	25.75	72.47	15.52
		60	0.826	23.70	71.18	13.93
		80	0.823	23.33	68.45	13.15

Table S9. Photovoltaic parameters of inverted OPV devices.

Active layer	V_{oc} [a] [V]	J_{sc} [a] [mA/cm^2]	Fill Factor [a] [%]	PCE [a] [%]
PM6:BTP-eC9	0.816 (0.810 ± 0.006)	21.52 (20.34 ± 1.18)	68.91 (66.28 ± 2.63)	12.09 (10.79 ±1.30)
PM6:BTP-eC9-γ	0.842 (0.837 ± 0.005)	21.72 (20.59 ± 1.13)	67.70 (65.79 ± 1.91)	12.39 (11.44 ± 0.95)

[a] the average device values are obtained from 15 cells.

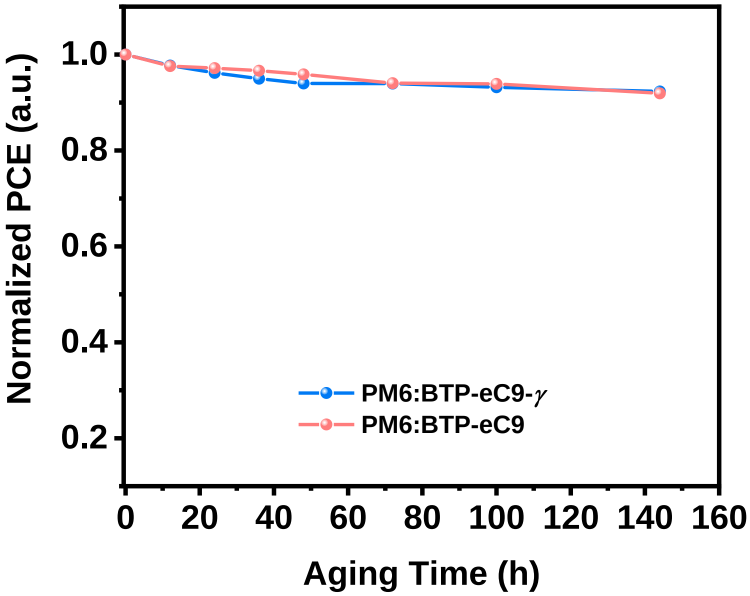

Figure S18. Normalized PCE for long-term stability test under illumination.

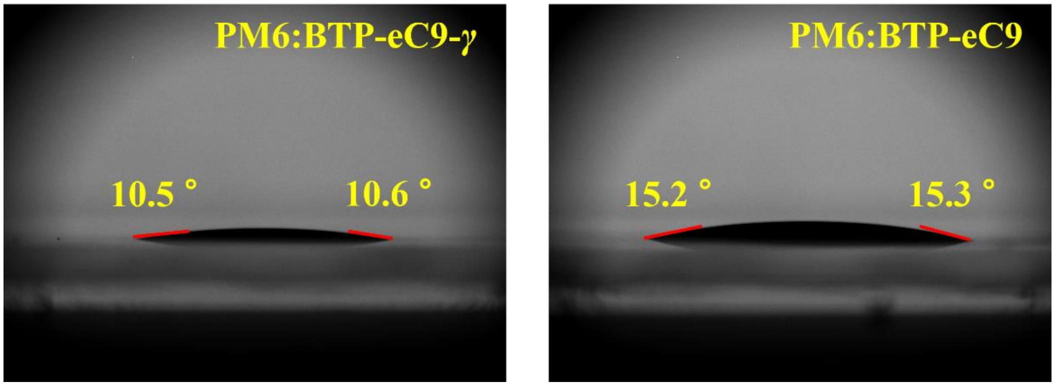

Figure S19. Contact angle measurements of PM6:BTP-eC9-γ and PM6:BTP-eC9. Each blend solution was dropped onto a PEDOT:PSS-coated ITO glass.
